# Pan-cancer integrated analysis of ANKRD1 expression, prognostic value, and potential implications in cancer

**DOI:** 10.1038/s41598-024-56105-2

**Published:** 2024-03-04

**Authors:** Xusan Xu, Dan Zhong, Xiaoxia Wang, Fei Luo, Xiaomei Zheng, Taoshan Feng, Riling Chen, Yisen Cheng, Yajun Wang, Guoda Ma

**Affiliations:** 1 Institute of Maternal and Child Research, Shunde Women and Children Hospital, Guangdong Medical University, Foshan, 528300 China; 2 Department of Neurology, Longjiang Hospital, Foshan, 528300 China; 3 Department of Pediatrics, Shunde Women and Children Hospital, Guangdong Medical University, Foshan, 528300 China; 4 Institute of Children’s Respiratory Diseases, Shunde Women and Children Hospital, Guangdong Medical University, Foshan, 528300 China

**Keywords:** Cancer, Computational biology and bioinformatics, Biomarkers, Oncology

## Abstract

There is substantial evidence demonstrating the crucial role of inflammation in oncogenesis. ANKRD1 has been identified as an anti-inflammatory factor and is related to tumor drug resistance. However, there have been no studies investigating the prognostic value and molecular function of ANKRD1 in pan-cancer. In this study, we utilized the TCGA, GTEx, GSCALite, ENCORI, CTRP, DAVID, AmiGO 2, and KEGG databases as well as R language, to explore and visualize the role of ANKRD1 in tumors. We employed the ROC curve to explore its diagnostic significance, while the Kaplan–Meier survival curve and Cox regression analysis were used to investigate its prognostic value. Additionally, we performed Pearson correlation analysis to evaluate the association between ANKRD1 expression and DNA methylation, immune cell infiltration, immune checkpoints, TMB, MSI, MMR, and GSVA. Our findings indicate that ANKRD1 expression is dysregulated in pan-cancer. The ROC curve revealed that ANKRD1 expression is highly sensitive and specific in diagnosing CHOL, LUAD, LUSC, PAAD, SKCM, and UCS (AUC > 85.0%, P < 0.001). Higher ANKRD1 expression was related to higher overall survival (OS) in LGG, but with lower OS in COAD and STAD (P < 0.001). Moreover, Cox regression and nomogram analyzes suggested that ANKRD1 is an independent factor for COAD, GBM, HNSC, and LUSC. Dysregulation of ANKRD1 expression in pan-cancer involves DNA methylation and microRNA regulation. Using the CTRP database, we discovered that ANKRD1 may influence the half-maximal inhibitory concentration (IC50) of several anti-tumor drugs. ANKRD1 expression showed significant correlations with immune cell infiltration (including cancer-associated fibroblast and M2 macrophages), immune checkpoints, TMB, MSI, and MMR. Furthermore, ANKRD1 is involved in various inflammatory and immune pathways in COAD, GBM, and LUSC, as well as cardiac functions in HNSC. In vitro experiments demonstrated that ANKRD1 promotes migration, and invasion activity, while inhibiting apoptosis in colorectal cancer cell lines (Caco2, SW480). In summary, ANKRD1 represents a potential prognostic biomarker and therapeutic target in human cancers, particularly in COAD.

## Introduction

Cancer is one of the major threats to human life and a severe global public health issue^1^. Further research has revealed the close involvement of immunity and inflammation in the pathogenesis of tumors and their influence on the response to anti-tumor therapy^2^. While emerging anti-cancer strategies, such as immune checkpoint blockade (ICB) therapy, are promising, a significant number of cancer patients still exhibit poor reactions to currently available therapies^3^. Thus, it remains critical to discover new diagnostic biomarkers and therapeutic targets for cancers.

Ankyrin Repeat Domain 1 (ANKRD1, also known as Cardiac Ankyrin Repeat Protein or Cardiac Adriamycin Responsive Protein, belongs to the muscle ankyrin repeat protein (MARP) family^4,5^. ANKRD1 exhibits specific expression in myocardial cells and plays a crucial role in myofibrillar assembly, signal transduction, transcriptional regulation, and maintenance of myocardial structure^4,6^. Previous studies have reported varied expression levels of ANKRD1 mRNA in different tumor cell lines, such as hepatoma^7^, and ovarian serous cystadenocarcinoma (OV)^8^. Additionally, overexpression of ANKRD1 has been associated with unfavorable outcomes in patients with OV^8^ and linked to resistance to cisplatin^9^ as well as second and third-generation epidermal growth factor receptor tyrosine kinase inhibitors (EGFR-TKIs)^10^. ANKRD1 is involved in the Hippo/YAP signaling pathway, which is associated with various cancers^11^. Moreover, it can interact with the tumor suppressor protein p53 and modulate its transcriptional activity^12^. Therefore, we speculate that ANKRD1 may play an important role in cancer etiology.

In this study, we performed a systematic pan-cancer (including 33 different types of cancer) analysis to explore the expression profile, diagnostic value, prognostic value, microRNA (miRNA) regulation, drug sensitivity of ANKRD1, and the potential relationship between ANKRD1 expression and tumor stage, DNA methylation level, mutation status, immunological function, and enriched pathways. In addition, we utilized in vitro models to evaluate the impact of ANKRD1 on the proliferation, migration, and invasion of colon adenocarcinoma (COAD).

## Results

### Gene expression analysis of ANKRD1 in human pan-cancer

**Figure 1:**
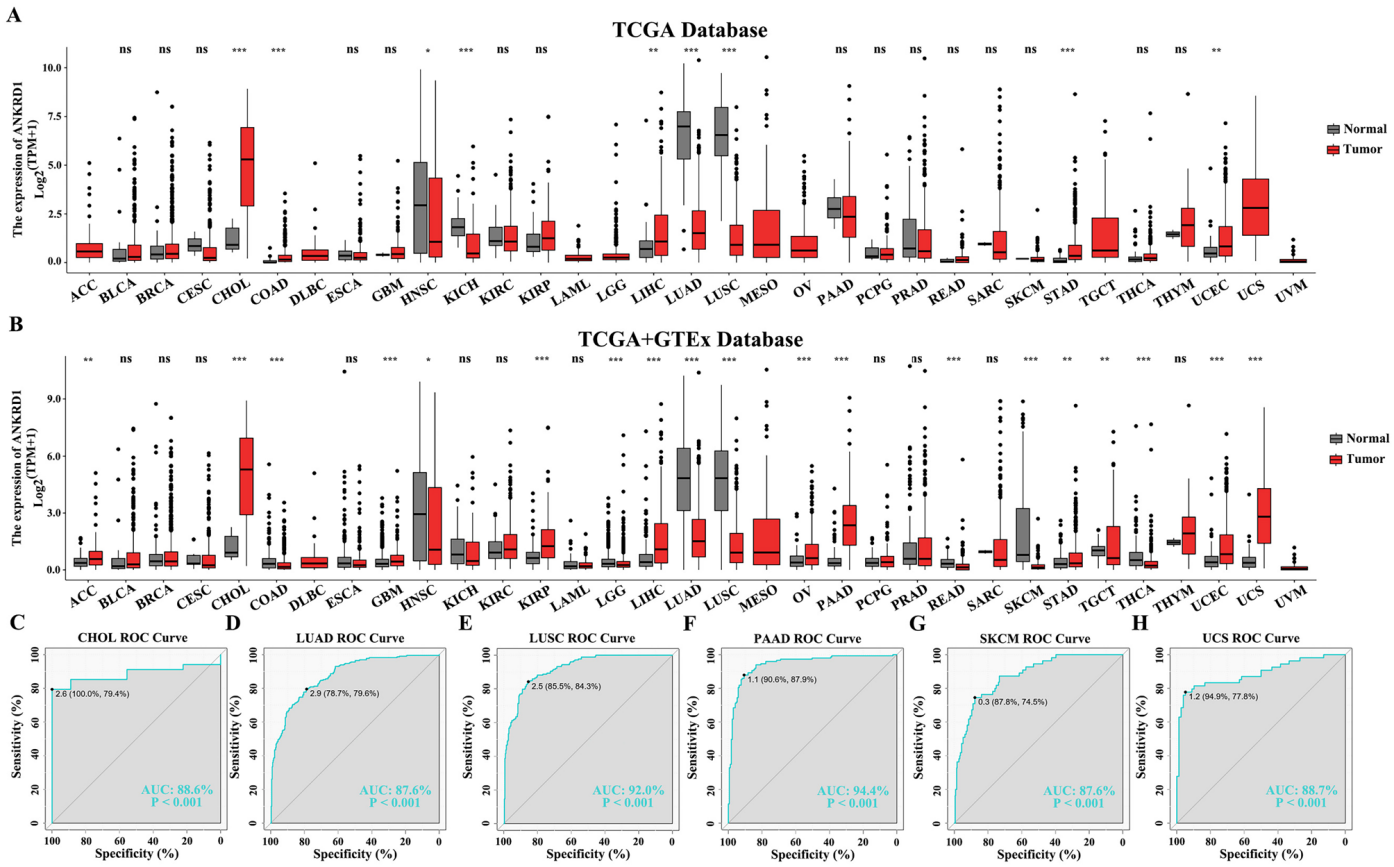
ANKRD1 mRNA expression levels in pan-cancer. (**A**) ANKRD1 mRNA expression levels in cancer and adjacent normal tissues in human pan-cancer from The Cancer Genome Atlas (TCGA) database. (**B**) ANKRD1 expression in cancer and normal tissues in human pan-cancer from TCGA and Genotype Tissue-Expression (GTEx) databases. (**C**–**H**) The receiver-operating characteristic (ROC) curve showed the high-expression specificity of ANKRD1 in CHOL, LUAD, LUSC, PAAD, SKCM, and UCS in the CGGA and TCGA databases. AUC, the area under the curve. *P < 0.05; **P < 0.01; ***P < 0.001, ns: no significance.

Compared to adjacent normal tissues, ANKRD1 mRNA expression was significantly increased in cholangiocarcinoma (CHOL), COAD, liver hepatocellular carcinoma (LIHC), stomach adenocarcinoma (STAD), and uterine corpus endometrial carcinoma (UCEC), but decreased in head and neck squamous cell carcinoma (HNSC), kidney Chromophobe (KICH), lung adenocarcinoma (LUAD), and lung squamous cell carcinoma (LUSC) tissues based on data from the Cancer Genome Atlas (TCGA) database (Fig. 1A). Considering the limited number of normal tissues in the TCGA database, we added the data from the genotype-tissue expression (GTEx) database. Most of the results are consistent with the previous findings. After combining both datasets, ANKRD1 was found to have significantly higher mRNA expression in adrenocortical carcinoma (ACC), CHOL, glioblastoma multiforme (GBM), kidney renal papillary cell carcinoma (KIRP), LIHC, OV, pancreatic adenocarcinoma (PAAD), STAD, UCEC, and uterine carcinosarcoma (UCS), but lower mRNA expression in COAD, HNSC, brain lower grade glioma (LGG), LUAD, LUSC, rectum adenocarcinoma (READ), skin cutaneous melanoma (SKCM), testicular germ cell tumor (TGCT), and thyroid carcinoma (THCA) (Fig. 1B). While ANKRD1 exhibited distinct expression levels between carcinoma and normal tissues, its expression remained stable across different clinical stages of pan-cancer, except for breast invasive carcinoma (BRCA), cervical squamous cell carcinoma (CESC), and KIRP (P < 0.05, Fig. S1).

To evaluate the expression specificity of ANKRD1 in pan-cancer, we conducted a receiver-operating characteristic (ROC) curve analysis. Our findings revealed that the area under the curve (AUC) was greater than 85.0% in CHOL, LUAD, LUSC, PAAD, SKCM, and UCS (P < 0.001) (Fig. 1C–H). These results suggest that ANKRD1 has the potential to serve as a diagnostic biomarker in CHOL, LUAD, LUSC, PAAD, SKCM, and UCS.

### Prognostic value of ANKRD1 in human pan-cancer

**Figure 2:**
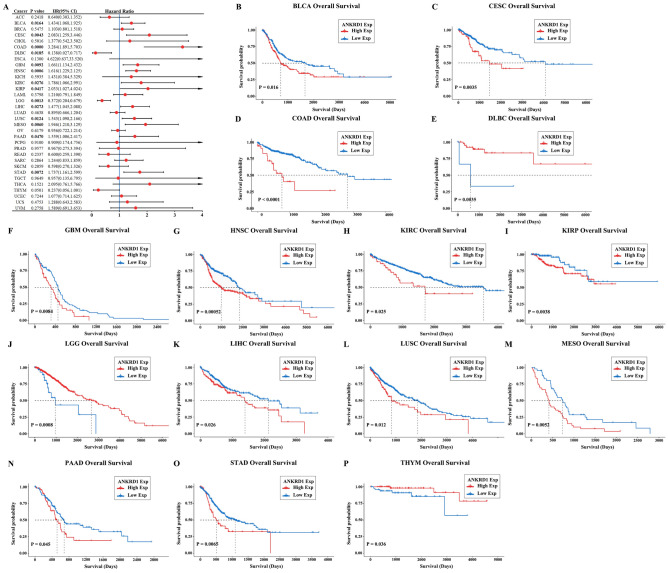
Survival analysis comparing the high and low expression of ANKRD1 on overall survival (OS) in pan-cancer. (**A**) Forest plot showing the impact of high expression of ANKRD1 on OS across 33 types of cancer. The significance of the prognostic value was assessed using Cox regression analysis. CI, confidence interval; HR, hazard ratio. (**B**–**H**) Kaplan–Meier analysis of ANKRD1 expression in various cancer types.

Cox regression analysis found that ANKRD1 expression was related to overall survival (OS) in 14 types of cancer, including bladder urothelial carcinoma (BLCA), CESC, COAD, diffuse large B-cell lymphoma (DLBC), GBM, HNSC, KIRC, KIRP, LGG, LIHC, LUSC, mesothelioma (MESO), PAAD, and STAD (P < 0.05) (Fig. 2A). The Kaplan–Meier (K-M) survival curves suggested that higher ANKRD1 expression was correlated with better OS in DLBC, LGG, and THYM, but with lower OS in BLCA, CESC, COAD, GBM, HNSC, kidney renal clear cell carcinoma (KIRC), KIRP, LIHC, LUSC, MESO, PAAD, and STAD (P < 0.05) (Fig. 2B–P).

### ANKRD1 as an independent prognostic factor in COAD, GBM, HNSC, LUSC, and STAD

**Figure 3:**
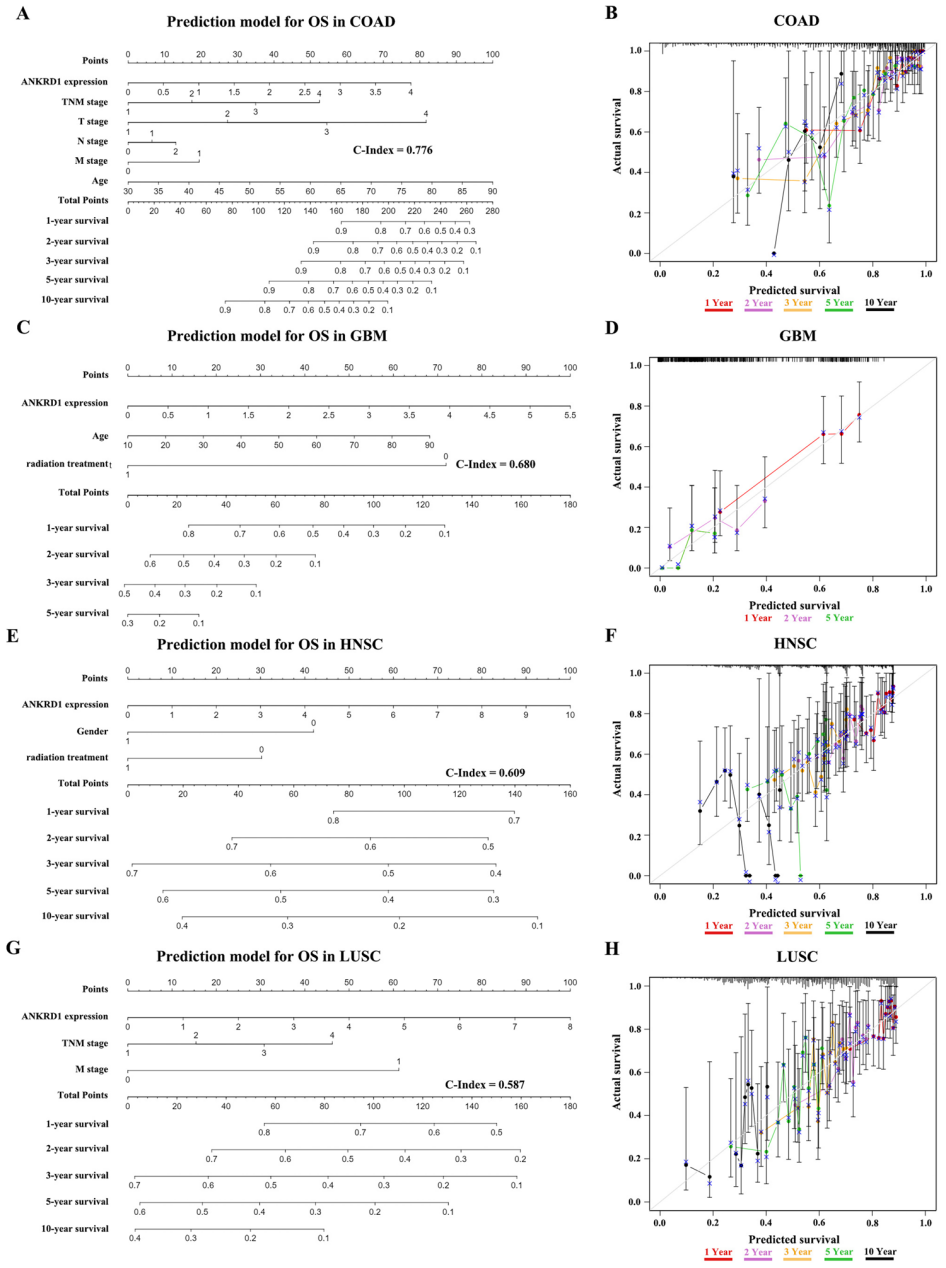
The prediction models for overall survival (OS) in COAD, GBM, HNSC, and LUSC. (**A**,**C**,**E**,**G**) The 1-, 2- 3-, 5-, and 10-year OS of patients with COAD, GBM, HNSC, or LUSC could be predicted by the nomogram. The predictive performance of the model on OS was evaluated using the C-Index. (**B**,**D**,**F**,**H**) Calibration plots were generated to compare the predicted OS with the actual OS at 1-, 2- 3-, 5-, and 10 years in COAD, GBM, HNSC, and LUSC.

To ascertain whether ANKRD1 is an independent prognostic factor in cancer, both univariate and multivariate Cox regression analysis were conducted. The results suggested that ANKRD1 expression was an independent prognostic factor in COAD, GBM, HNSC, LUSC, and STAD, independent of known prognostic factors, such as TNM stage, gender, age, pharmaceutical treatment, and radiation treatment (P < 0.05) (Tables 1). In addition, the nomogram was established based on multivariate analysis (Fig. 3A,C,E,G). The C-index and calibration curve were further used to confirm the accuracy in predicting the 1-, 2-, 3-, 5-, and 10-years overall survival in COAD, GBM, HNSC, and LUSC (C-index = 0.776, 0.680, 0.609, and 0.587, respectively) (Fig. 3B,D,F,H).

**Table 1:** Univariate and multivariate analysis of prognostic parameters in the TCGA database for overall survival.

Cancer	Variable	Univariate analysis		Multivariate analysis	
		P-value	HR (95% CI)	P-value	HR (95% CI)
COAD	ANKRD1 expression	0.013	1.544 (1.097, 2.172)	0.029	1.599 (1.050, 2.434)
	TNM stage	5.26E−09		8.19E−06	
	T stage	1.15E−05		0.028	
	N stage	1.70E−08		0.020	
	M stage	4.03E−10	4.175 (2.667, 6.534)	–	
	Gender	0.659	1.092 (0.738, 1.615)		
	Age	1.89E−03	1.028 (1.010, 1.046)	3.47E−05	1.044 (1.023, 1.066)
	Pharmaceutical treatment	0.558	1.148 (0.724, 1.818)		
	Radiation treatment	0.616	0.697 (0.170, 2.854)		
GBM	ANKRD1 expression	0.011	1.402 (1.081, 1.817)	0.003	1.479 (1.139, 1.920)
	Gender	0.037	1.217 (1.012, 1.464)	0.103	1.460 (0.926, 2.301)
	Age	1.80E−21	1.035 (1.028, 1.042)	0.015	1.018 (1.004, 1.033)
	Pharmaceutical treatment	1.74E−18	0.289 (0.219, 0.382)	0.380	1.592 (0.564, 4.497)
	Radiation treatment	5.90E−33	0.197 (0.151, 0.257)	3.12E−05	0.105 (0.036, 0.303)
HNSC	ANKRD1 expression	0.017	1.062 (1.011, 1.116)	2.78E−04	1.238 (1.103, 1.389)
	TNM stage	7.64E−04		0.339	
	T stage	0.001		0.720	
	N stage	3.74E−07		0.089	
	M stage	0.004	20.279 (2.569, 160.072)	0.125	8.287 (0.558, 123.134)
	Gender	0.023	0.721 (0.544, 0.956)	0.023	0.490 (0.265, 0.907)
	Age	0.001	1.021 (1.008, 1.033)	0.300	1.013 (0.989,1.038)
	Pharmaceutical treatment	0.627	1.078 (0.795, 1.463)		
	Radiation treatment	0.046	0.730 (0.536, 0.994)	2.97E−04	0.265 (0.129, 0.544)
LUSC	ANKRD1 expression	0.005	1.149 (1.042, 1.268)	0.009	1.172 (1.040, 1.320)
	TNM stage	0.011		0.043	
	T stage	0.008		0.477	
	N stage	0.404			
	M stage	0.014	3.064 (1.252, 7.495)	–	
	Gender	0.267	1.197 (0.871, 1.645)		
	Age	0.031	1.018 (1.002, 1.035)		
	Pharmaceutical treatment	0.367	0.864 (0.628, 1.187)		
	Radiation treatment	0.037	1.455 (1.022, 2.071)	0.381	1.217 (0.784, 1.888)
STAD	ANKRD1 expression	0.005	1.223 (1.064, 1.407)	2.76E−04	1.350 (1.148, 1.587)
	TNM stage	7.38E−06		0.125	
	T stage	0.025		0.919	
	N stage	6.18E−05		0.275	
	M stage	2.22E−04	2.585 (1.562, 4.279)	0.395	0.652 (0.243, 1.749)
	Gender	0.659	1.074 (0.782, 1.475)		
	Age	0.007	1.020 (1.005, 1.036)	0.108	1.016 (0.996, 1.036)
	Pharmaceutical treatment	0.010	0.659 (0.480, 0.904)	0.192	0.748 (0.484, 1.156)
	Radiation treatment	1.69E−04	0.430 (0.277, 0.667)	0.055	0.579 (0.332, 1.011)

CI, confidence interval; HR, hazard ratio.

### DNA methylation analysis of ANKRD1 in pan-cancer

**Figure 4:**
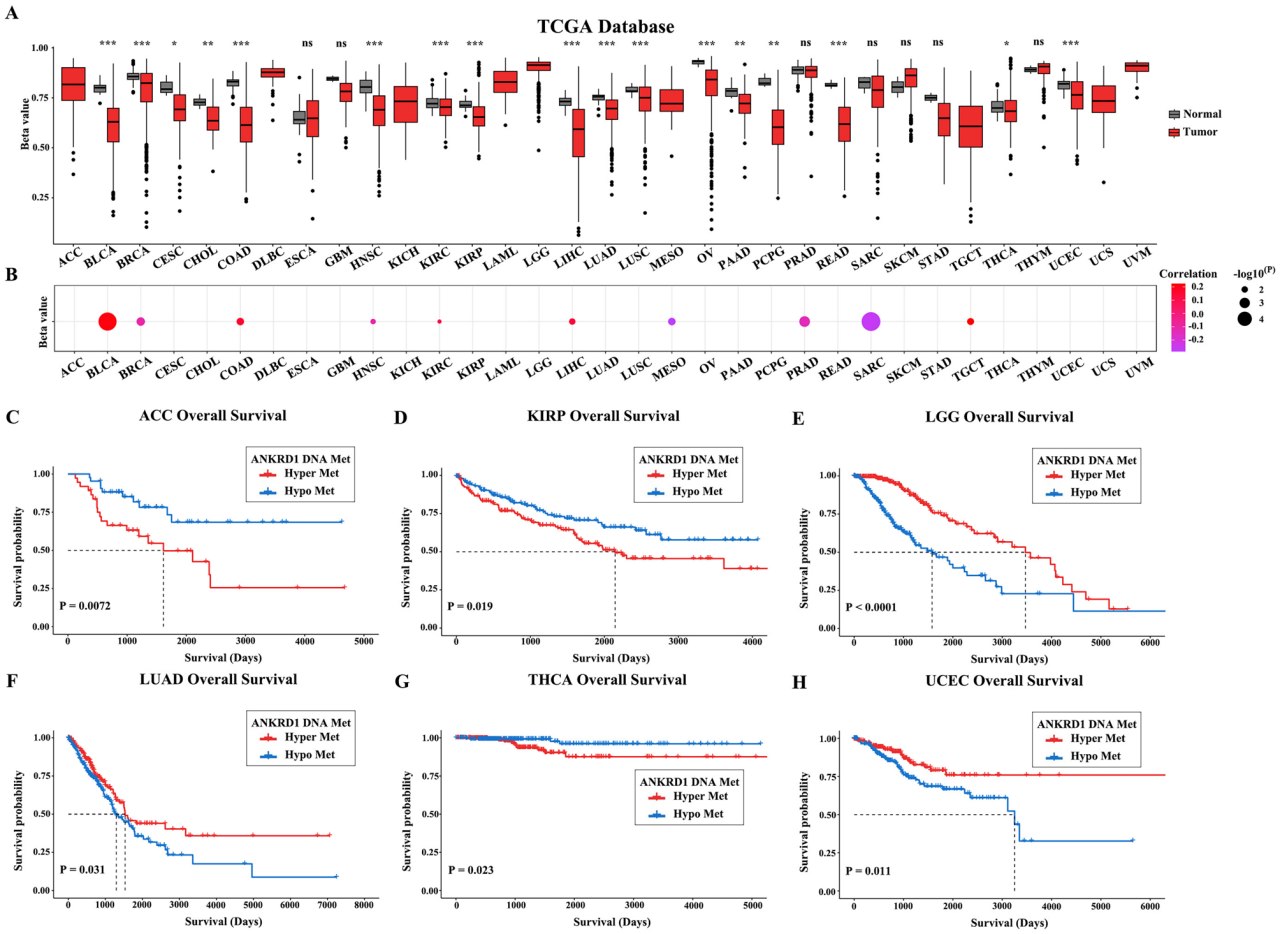
DNA methylation level of ANKRD1 in pan-cancer. (**A**) The DNA methylation level of ANKRD1 in cancer and adjacent normal tissues in human pan-cancer from TCGA database. (**B**) Correlation between DNA methylation and mRNA expression of ANKRD1. Purple points represent a negative correlation and red points represent a positive correlation. (**C**–**H**) Kaplan–Meier analysis of the DNA methylation level of ANKRD1 in ACC, KIRC, LGG, LUAD, THCA, and UCEC in the TCGA databases. *P < 0.05; **P < 0.01; ***P < 0.001, ns: no significance.

The level of DNA methylation can affect gene expression and alterations in DNA methylation of many genes have been observed in various tumors. Here, we found a significant decrease in the DNA methylation level of ANKRD1 in BLCA, BRCA, CESC, CHOL, COAD, HNSC, KIRC, KIRP, LIHC, LUAD, LUSC, OV, PAAD, pheochromocytoma and paraganglioma (PCPG), READ, THCA, and UCEC based on the TCGA database (Fig. 4A). Furthermore, we found that the DNA methylation level of ANKRD1 was positively related to ANKRD1 expression in BLCA, COAD, KIRC, LIHC, and TGCT, but negatively associated with ANKRD1 expression in BRCA, HNSC, MESO, prostate adenocarcinoma (PRAD), and sarcoma (SARC) (Fig. 4B). Additionally, the K-M survival curves suggested that hyper-methylation of ANKRD1 was associated with better OS in LGG, LUAD, and UCEC, but with worse OS in ACC, KIRP, and THCA (Fig. 4C–H).

### Somatic mutations and copy number variation (CNV) of ANKRD1 in pan-cancer

**Figure 5:**
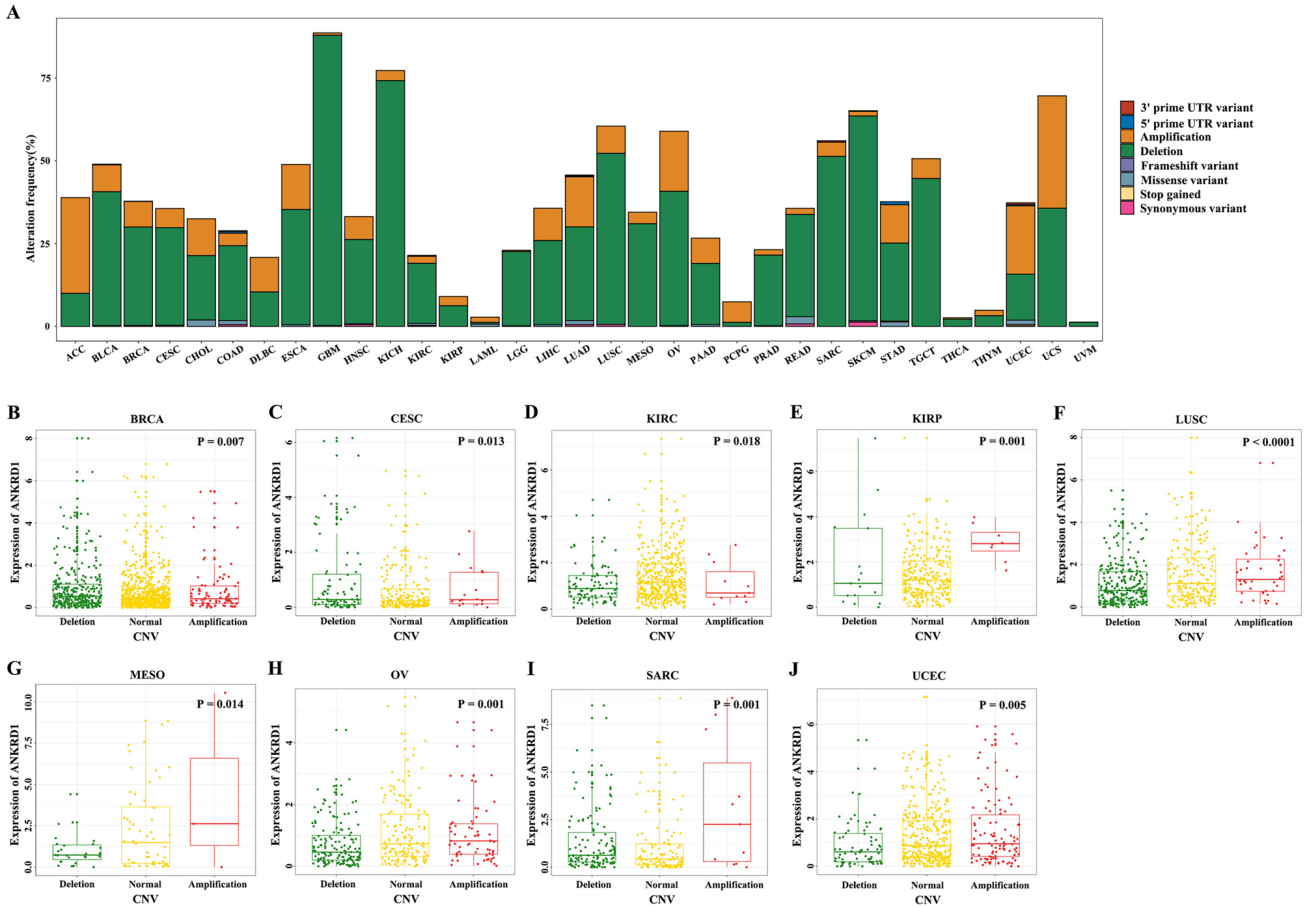
Somatic mutations and copy number variation (CNV) of ANKRD1 in Pan-Cancer. (**A**) The alteration frequency of ANKRD1 with different types of single nucleotide variation (SNV) and CNV was obtained from the TCGA database. (**B**–**J**) The relationship between ANKRD1 mRNA expression and CNV subtypes (deletion, normal, and amplification copy) in BRCA, CESC, KIRC, KIRP, LUSC, MESO, OV, SARC, and UCEC.

As shown in Fig. 5A, missense mutations were the primary type of single nucleotide variation (SNV) and depletion was the predominant type of CNV in ANKRD1. Moreover, we observed significant differences in ANKRD1 mRNA expression among the deletion, normal, and amplification copy of ANKRD1 in BRCA, CESC, KIRC, KIRP, LUSC, MESO, OV, SARC, and UCEC (Fig. 5B–J).

### miRNA regulation analysis

**Figure 6:**
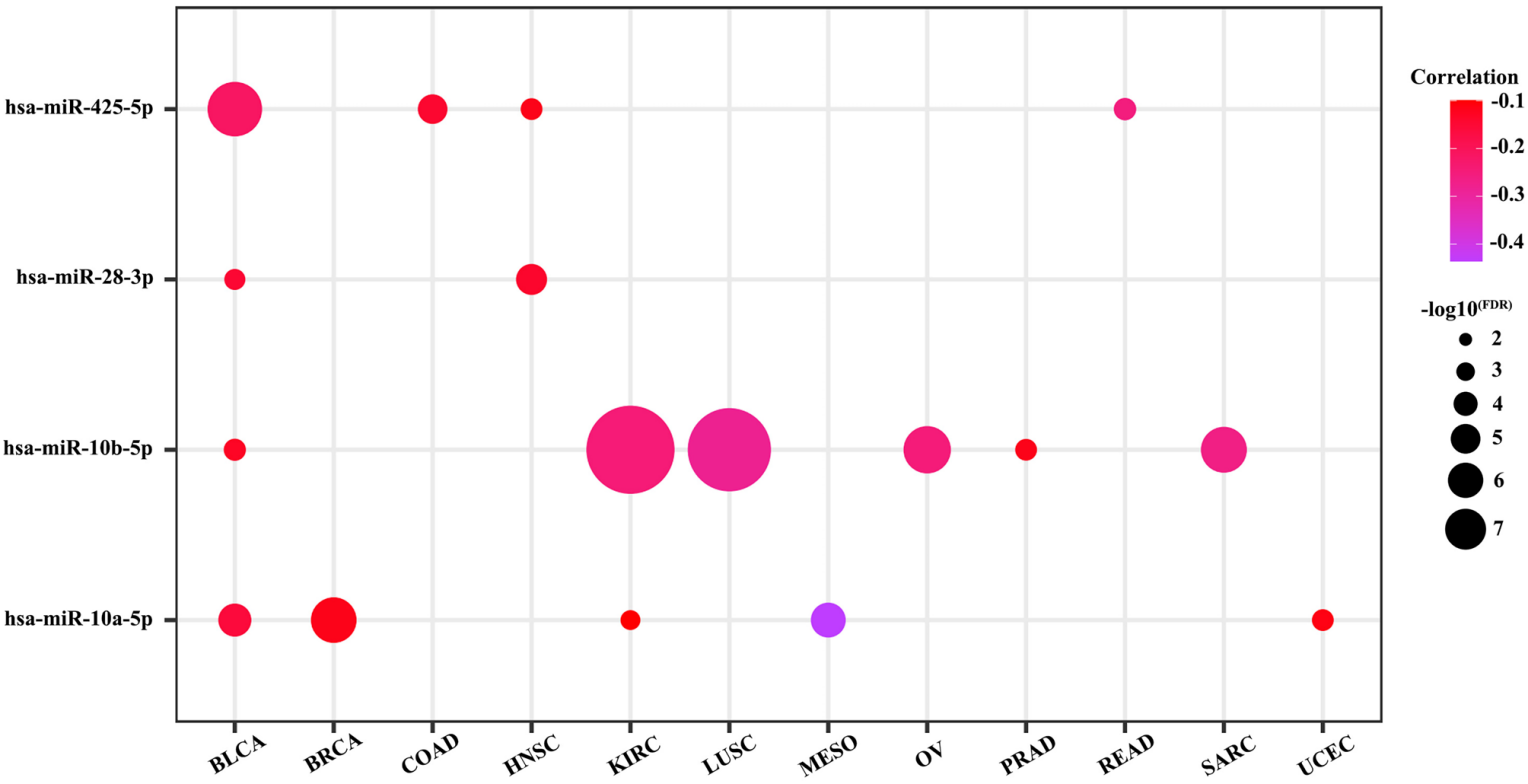
Correlation between miRNA and ANKRD1 mRNA expression. The darker the color, the higher the correlation. FDR, false discovery rate.

The role of miRNA in gene expression regulation has been well-established. As shown in Fig. 6, miR-10a-5p negatively regulated ANKRD1 expression in BLCA, BRCA, KIRC, MESO, and UCEC, miR-10b-5p negatively regulated ANKRD1 expression in BLCA, KIRC, LUSC, OV, PRAD, and SARC, miR-28-3p negatively regulated ANKRD1 expression in BLCA, and HNSC, miR-425-5p negatively regulated ANKRD1 expression in BLCA, COAD, HNSC, and READ.

### Drug sensitivity analysis of ANKRD1

**Figure 7:**
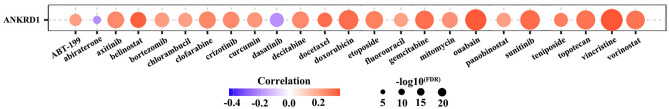
Drug sensitivity analysis of ANKRD1. ANKRD1 drug resistance analysis from CTRP database. Spearman correlation analysis was used to investigate the correlation between ANKRD1 mRNA level and drugs. The darker the color, the higher the correlation. FDR, false discovery rate.

To explore the impact of ANKRD1 on chemotherapy or targeted therapy, we collected data from the Cancer Therapeutics Response Portal (CTRP) database, which includes drug sensitivity and ANKRD1 mRNA expression information from various cancer cell lines. Spearman correlation analysis suggested that the expression of ANKRD1 negatively correlated with the IC50 (half maximal inhibitory concentration) of dasatinib, and abiraterone, but positively correlated with the IC50 of vorinostat, vincristine, topotecan, teniposide, sunitinib, panobinostat, ouabain, mitomycin, gemcitabine, fluorouracil, etoposide, doxorubicin, docetaxel, decitabine, curcumin, crizotinib, clofarabine, chlorambucil, bortezomib, belinostat, axitinib, and ABT-199 (Fig. 7).

### Association between ANKRD1 expression and immune cell infiltration in pan-cancer

**Figure 8:**
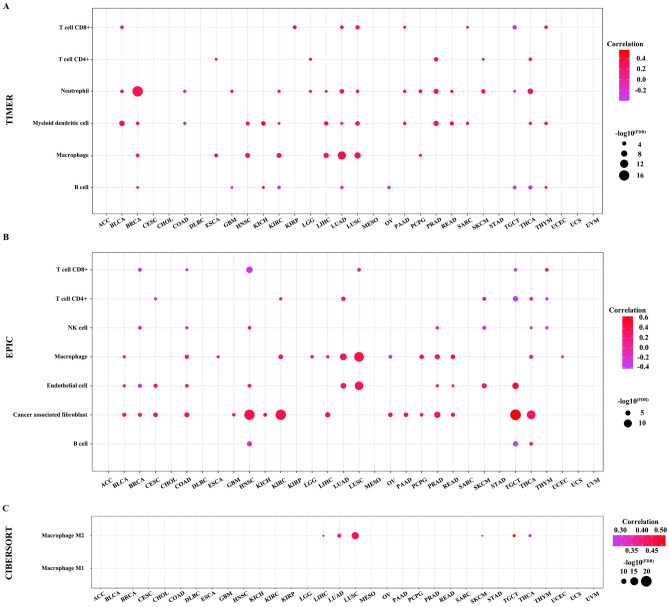
Correlation between the infiltration level of immune cell and ANKRD1 mRNA expression. (**A**) Bubble chart showed the correlation between ANKRD1 and immune infiltration using TIMER algorithm. (**B**) Bubble chart displayed the correlation between ANKRD1 and immune infiltration under EPIC algorithm. The darker the color, the higher the correlation. FDR, false discovery rate.

To investigate whether ANKRD1 affects immune infiltration, we utilized the TIMER, EPIC, and CIBERSORT algorithm to estimate the association between ANKRD1 gene expression and immune cell infiltration levels. According to the TIMER algorithm, ANKRD1 expression exhibited a positive correlation with macrophage infiltrating level in LIHC, LUAD, and LUSC, myeloid dendritic cell in BLCA, and PRAD, and neutrophil in BRCA, PRAD, and THCA (Fig. 8A, |R| > 0.25, −log10^(FDR)^ > 5). The EPIC algorithm showed that ANKRD1 expression was positively correlated with the infiltrating level of cancer-associated fibroblast in COAD, HNSC, KIRC, LIHC, PRAD, TGCT, and THCA, endothelial cell in LUAD, LUSC, and TGCT, macrophage in LUAD and LUSC, but negatively correlated with the infiltrating level of B cell in TGCT, CD4^+^ T cell in TGCT, and CD8^+^ T cell in HNSC (Fig. 8B, |R| > 0.25, −log10^(FDR)^ > 5). Furthermore, using the CIBERSORT algorithm for macrophage, we observed a positive correlation between ANKRD1 expression and the infiltrating level of M2 macrophage in LIHC, LUAD, LUSC, SKCM, TGCT, and THCA (Fig. 8C, |R| > 0.25, −log10^(FDR)^ > 5).

### Relationship between ANKRD1 expression and immune checkpoints, TMB, MSI, and MMR

**Figure 9:**
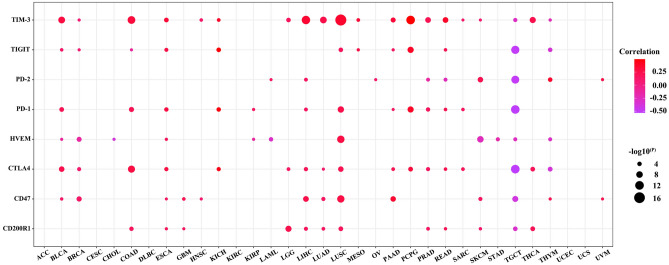
Correlation between immune checkpoint genes and ANKRD1 mRNA expression. The darker the color, the higher the correlation.

As shown in Fig. 9, significant relationships were observed between ANKRD1 and immune checkpoint genes, such as including CD200R1, CD47, cytotoxic T lymphocyte associated protein 4 (CTLA4), herpes virus entry mediator A (HVEM), programmed death-1 (PD-1), PD-2, TIGIT, and TIM-3, in most cancers except ACC, CESC, DLBC, KIRC, UCEC, and UCS. Moreover, the expression of ANKRD1 positively correlated with immune checkpoint genes in most cancers, except TGCT (Fig. 9).

**Figure 10:**
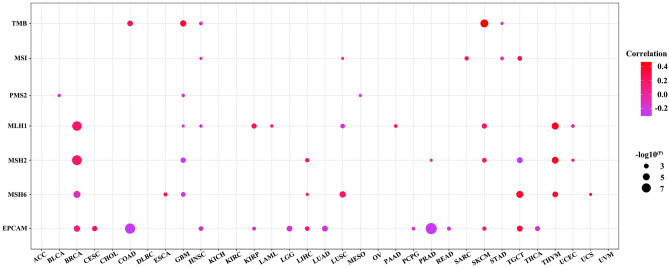
Correlation between ANKRD1 mRNA expression and TMB, MSI, and MMR. The darker the color, the higher the correlation.

Tumor mutation burden (TMB), microsatellite instability (MSI), and mismatch repair (MMR) are related to the immunotherapy response. We found that ANKRD1 expression was positively correlated with TMB in COAD, GBM, and SKCM, but negatively correlated with TMB in HNSC and STAD (Fig. 10). Additionally, our results suggested that ANKRD1 expression was positively correlated with MSI in SARC and TGCT, but negatively correlated with MSI in HNSC, LUSC, and STAD (Fig. 10). When examining the relationship between ANKRD1 expression and essential MMR signatures (PMS1 homolog 2 (PMS2), MutL homolog 1 (MLH1), MutS homolog 2 (MSH2) and MutS homolog 6 (MSH6), and epithelial cell adhesion molecule (EPCAM)), significant associations were observed in most cancers, except ACC, CHOL, DLBC, KICH, KIRC, OV, SARC, STAD, and UVA (Fig. 10).

### GO and KEGG analyses of ANKRD1 in pan-cancer

**Figure 11:**
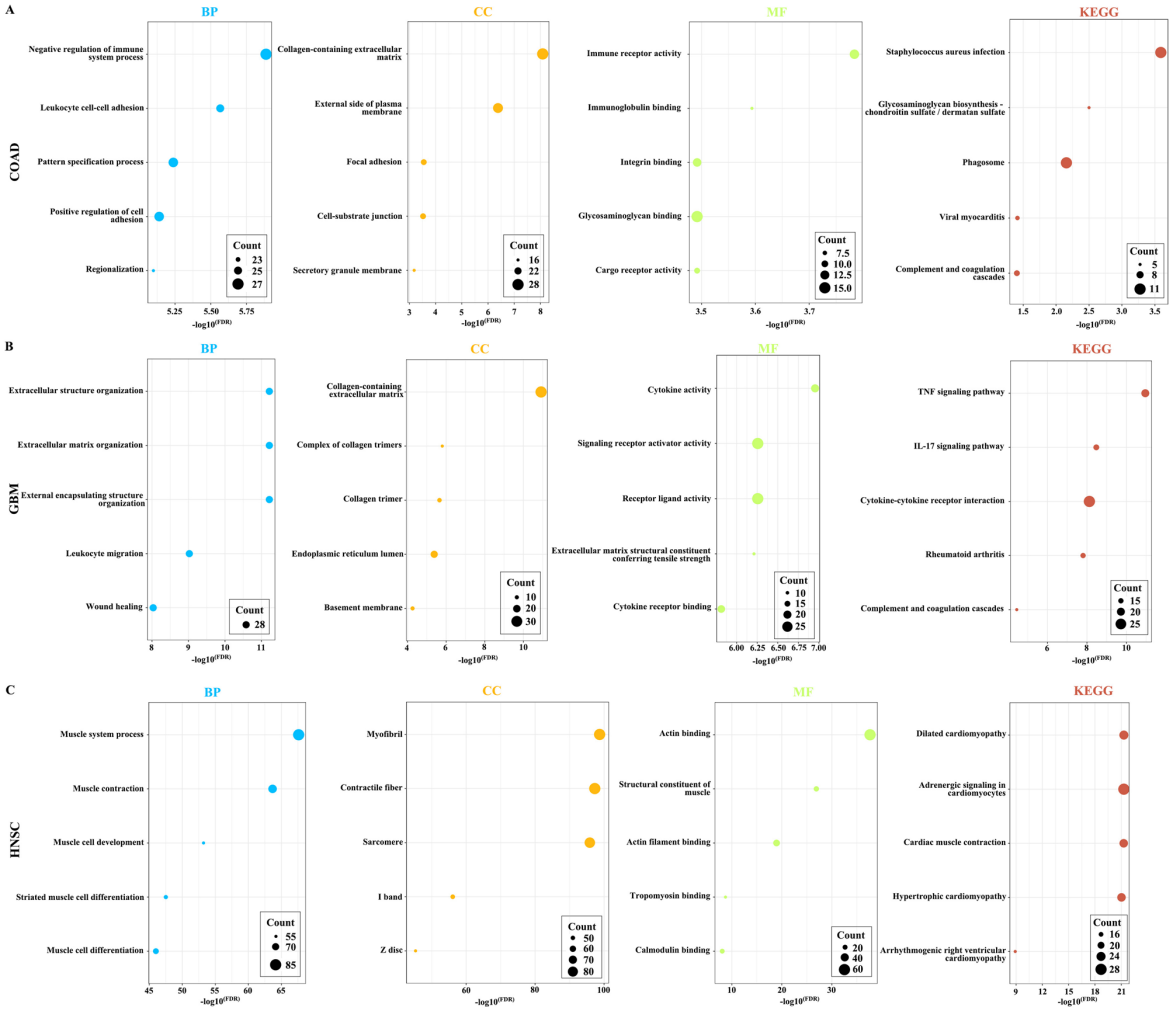
GO and KEGG (Retrieved from http://www.kegg.jp/kegg/kegg1.html) enrichment analyses for ANKRD1. Top 5 pathways enriched in the BP, CC, MF, and KEGG analyses in (**A**) COAD, (**B**) GBM, and (**C**) HNSC.

To investigate the biological functions related to ANKRD1 in cancers, the top 300 genes related to ANKRD1 were identified through Pearson correlation analysis based on the TCGA databases. Subsequently, Gene Ontology (GO) and Kyoto Encyclopedia of Genes and Genomes (KEGG) analysis were performed based on the above gene sets. The most enriched BP (biological process) were negative regulation of immune system process, leukocyte cell–cell adhesion, pattern specification process, positive regulation of cell adhesion, and regionalization in COAD; extracellular matrix organization, extracellular structure organization, external encapsulating structure organization, leukocyte migration, and wound healing in GBM; muscle system process, muscle contraction, muscle cell development, striated muscle cell differentiation, and muscle cell differentiation in HNSC; surfactant homeostasis, chemical homeostasis within a tissue, complement activation, alternative pathway, mesenchymal cell differentiation, and humoral immune response in LUSC; and acute-phase response, negative regulation of fibrinolysis, acute inflammatory response, detection of chemical stimulus involved in sensory perception of smell, and sensory perception of smell in STAD (Figs. 11A–C, S2A,B). Regarding cellular components (CC), the most enriched CC were collagen-containing extracellular matrix, external side of plasma membrane, focal adhesion, cell-substrate junction, and secretory granule membrane in COAD; collagen-containing extracellular matrix, complex of collagen trimers, collagen trimer, endoplasmic reticulum lumen, and basement membrane in GBM; myofibril, contractile fiber, sarcomere, I band, and Z disc in HNSC; lamellar body, multivesicular body, endocytic vesicle, clathrin-coated endocytic vesicle, and clathrin-coated vesicle in LUSC; and blood microparticle, P-body, and collagen-containing extracellular matrix in STAD (Figs. 11A–C, S2A,B). Furthermore, ANKRD1’s most related MFs (molecular functions) were immune receptor activity, immunoglobulin binding, glycosaminoglycan binding, cargo receptor activity, and integrin binding in COAD; cytokine activity, receptor ligand activity, signaling receptor activator activity, extracellular matrix structural constituent conferring tensile strength, and cytokine receptor binding in GBM; actin binding, structural constituent of muscle, actin filament binding, tropomyosin binding, and calmodulin binding in HNSC; cargo receptor activity, scavenger receptor activity, immune receptor activity, carbohydrate binding, and transmembrane receptor protein kinase activity in LUSC; and cysteine-type endopeptidase inhibitor activity in STAD (Figs. 11A–C, S2A,B).

Additionally, KEGG analysis found that ANKRD1 was associated with signaling pathways related to staphylococcus aureus infection, glycosaminoglycan biosynthesis—chondroitin sulfate/dermatan sulfate, phagosome, viral myocarditis, and complement and coagulation cascades in COAD; TNF signaling pathway, IL-17 signaling pathway, cytokine-cytokine receptor interaction, rheumatoid arthritis, and complement and coagulation cascades in GBM; dilated cardiomyopathy, adrenergic signaling in cardiomyocytes, cardiac muscle contraction, hypertrophic cardiomyopathy, and arrhythmogenic right ventricular cardiomyopathy in HNSC; complement and coagulation cascades in LUSC; and complement and coagulation cascades and olfactory transduction in STAD (Figs. 11A–C, S2A,B).

### Gene set variation analysis (GSVA) and Gene Set Enrichment Analysis (GSEA) of ANKRD1 in pan-cancer

**Figure 12:**
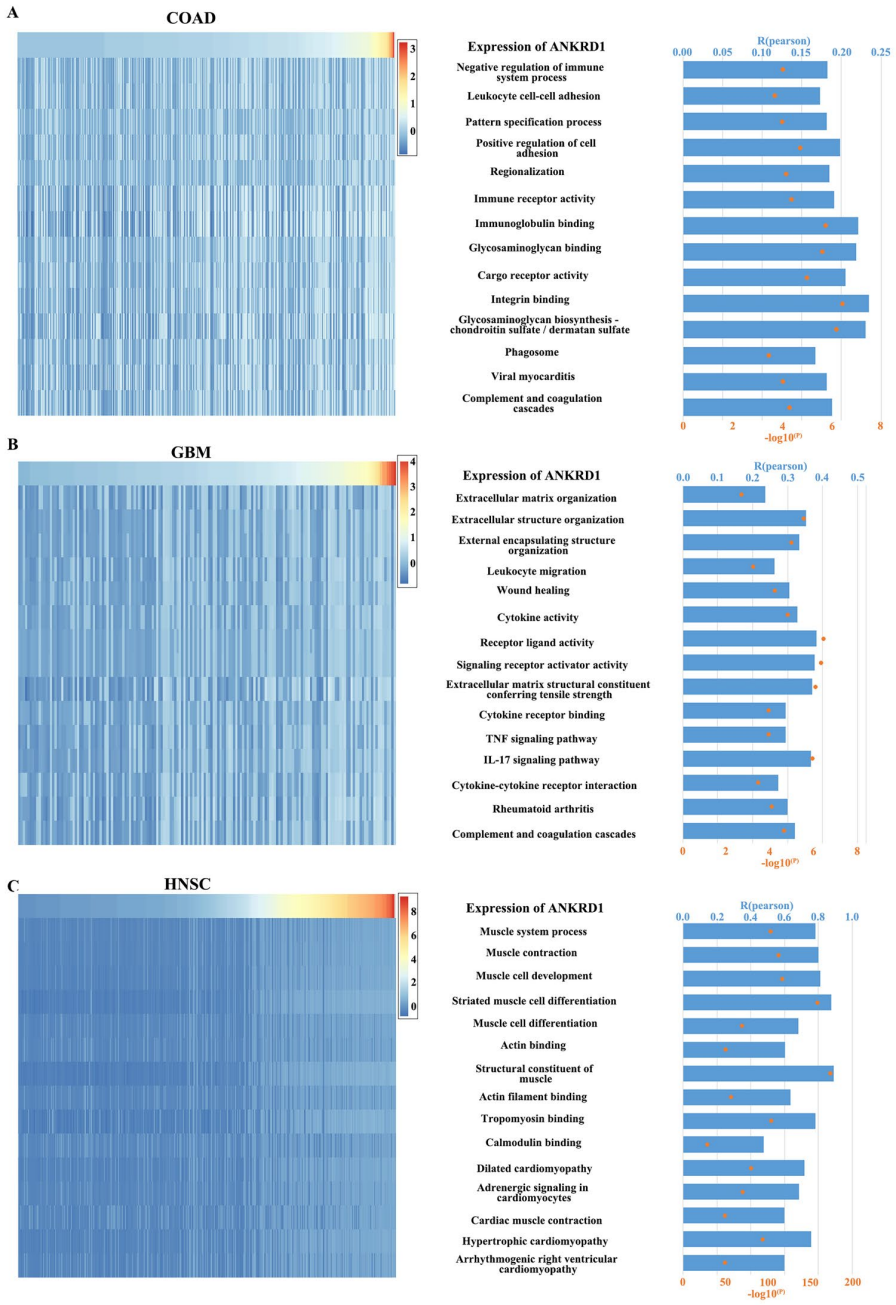
Correlation analysis between ANKRD1 expression and the enrichment scores of enriched pathways based on the BP, MF, and KEGG analyses in (**A**) COAD, (**B**) GBM, and (**C**) HNSC. The heatmap showed ANKRD1 mRNA expression and the enrichment scores of each patient in the TCGA database. The samples were arranged in ascending order of the expression of ANKRD1. The column graph and line graph on the right showed the R-value and P-value of the correlation analysis.

The related gene sets were obtained from the AmiGO 2 and KEGG database. Pearson correlation analysis was conducted to examine the relationship between the enrichment score and the expression of ANKRD1. The results indicated that ANKRD1 exhibited positive correlation with negative regulation of immune system process, immune receptor activity, leukocyte cell–cell adhesion, positive regulation of cell adhesion, regionalization, cargo receptor activity, integrin binding, phagosome, and complement and coagulation cascades in COAD; leukocyte migration, myeloid leukocyte migration, TNF signaling pathway, IL-17 signaling pathway, rheumatoid arthritis, and complement and coagulation cascades in GBM; muscle system process, muscle contraction, muscle cell development, striated muscle cell differentiation, muscle cell differentiation, actin binding, structural constituent of muscle, actin filament binding tropomyosin binding, calmodulin binding, dilated cardiomyopathy, adrenergic signaling in cardiomyocytes, cardiac muscle contraction, hypertrophic cardiomyopathy, and arrhythmogenic right ventricular cardiomyopathy in HNSC; complement activation, alternative pathway, humoral immune response, cargo receptor activity, scavenger receptor activity, immune receptor activity, and complement and coagulation cascades in LUSC; but fail to related the above enriched BP, MF, or signaling pathway (Figs. 12A–C, S3A,B). To validate the findings of the gene enrichment analysis, another algorithm (GSEA) was employed (Table S2). The majority of the results still showed significant differences (FDR < 0.05), except for acute-phase response, acute inflammatory response, and cysteine-type endopeptidase inhibitor activity in STAD. Taken together, these results suggest a potential linkage between ANKRD1 and inflammatory and immune responses in COAD, GBM, and LUSC; and cardiac functions in HNSC.

### ANKRD1 promotes migration, and invasion activity and inhibits apoptosis of colorectal cancer (CRC) cells

**Figure 13:**
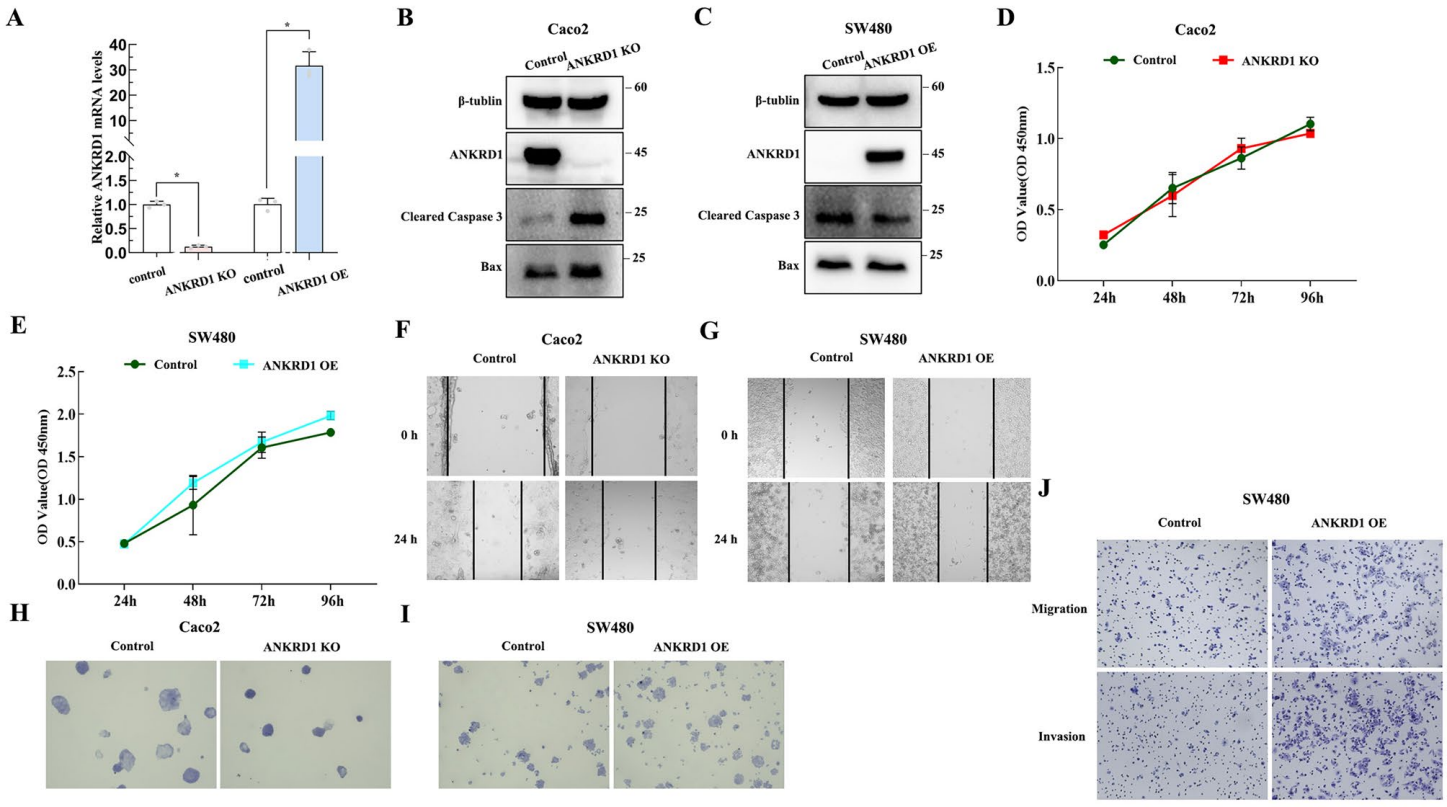
The biological functions of ANKRD1 in COAD. Knockout efficiency or over-expression efficiency of ANKRD1 proved by qPCR in Caco2 and SW480 cells, respectively (**A**); Knockout efficiency or over-expression efficiency of ANKRD1 proved by WB in Caco2 and SW480 cells, respectively (**B**,**C**); the biological functions of ANKRD1 on COAD cell lines were confirmed by apoptosis related proteins (**B**,**C**) (The membranes were cropped at indicated region specified in Supplementary Information), CCK-8 (**D**,**E**), scratch test (**F**,**G**), colony-formation assay (**H**,**I**) and transwell migration and invasion assay (**J**).

ANKRD1 hasn't been explored in COAD. Therefore, we utilized lentivirus to mediate ANKRD1 expression in Caco2 and SW480 cell lines to observe any changes in the biological functions of colorectal cancer cells. Figure 13A,B displayed the knockdown efficiency, showing that the ANKRD1 KO group exhibited a significant decrease in ANKRD1 expression or protein in Caco2 cells. Figure 13A,C showed the overexpression efficiency, indicating that the ANKRD1 OE group had a significant increase in ANKRD1 expression or protein in SW480 cells. We found that silencing ANKRD1 expression significantly increased the expression of apoptosis related proteins (Bax, cleared caspase 3), while overexpression of ANKRD1 inhibited the expression of these proteins (Fig. 13B,C). In the CCK-8 experiment conducted on the Caco2 cell line, the ANKRD1 KO group displayed similar growth rates over time when compared to the control group (Fig. 13D). Similarly, the ANKRD1 OE group in SW480 cells exhibited consistent results (Fig. 13E), suggesting that ANKRD1 does not significantly influence the proliferation of colorectal cancer cells. The colony-formation assay found that ANKRD1 KO decreased the tumorigenicity of CRC (COAD) cells (Fig. 13H), while ANKRD1 OE enhanced the tumorigenicity of CRC (SW480) cells (Fig. 13I). The scratch test results (Fig. 13F,G) suggested that ANKRD1 promotes the migration of CRC cells. The positive effect of ANKRD1 on CRC cells migration was further confirmed by the transwell migration assay (Fig. 13J). Moreover, the simultaneous transwell invasion assay conducted (Fig. 13J) also showed the stimulating effect of ANKRD1 on the invasion activity of CRC cells. We failed to conduct transwell migration and invasion assay on Caco2 cells as they were unable to penetrate the pores of the transwell chamber (date not shown).

## Discussion

Although great progress in the diagnosis and treatment of cancer with continuous advancement in the molecular mechanisms (including oncogenesis, metastasis, and so on), cancer remains a global public health challenge. Therefore, it is necessary to continuously find more susceptible diagnostic biomarkers and efficient therapeutic targets for cancer. ANKRD1, a multifunctional gene, has been mentioned in various tumors^8^ and is associated with tumor-related pathways^12,13^ and tumor resistance^9,10^. However, the role of ANKRD1 in pan-cancer has not been thoroughly investigated. Thus, in this study, we employed multiple bioinformatics approaches to comprehensively evaluate the potential roles of ANKRD1 in 33 different types of tumors in many aspects (expression level, prognostic value, epigenetics, mutation status, etc.) based on the TCGA, GTEx, GSCALite, ENCORI, CTRP, DAVID, AmiGO 2 and KEGG databases.

ANKRD1 exhibited distinct expression patterns in pan-cancer and was found to be abnormally expressed in 19 types of tumors based on TCGA + GTEx databases (P < 0.05, Fig. 1B). This suggests that ANKRD1 may have a significant role in tumor development and progression. Furthermore, the ROC curve analysis revealed that ANKRD1 serves as a sensitive and specific marker for CHOL, LUAD, LUSC, PAAD, SKCM, and UCS (AUC > 85.0%, P < 0.001) (Fig. 1C–H). This finding implies that ANKRD1 could potentially contribute to the differential diagnosis of these tumors. Interestingly, a previous study reported the diagnostic utility of ANKRD1 protein immunostaining in rhabdomyosarcoma^14^. However, our results based on mRNA levels did not show well discriminability (AUC = 36.4%, P > 0.05) (data not shown). This discrepancy could be partially attributed to the limited cases of the normal group, with only two normal tissues available in the SARC dataset. Furthermore, ANKRD1 mRNA levels were found to possess prognostic value in predicting overall survival (OS) for 14 types of cancers (Fig. 2). In particular, ANKRD1 emerged as an independent prognostic factor in COAD, GBM, HNSC, LUSC, and STAD (Fig. 3). Contradicting our findings, a retrospective cohort study involving 71 patients with OV from Australia suggested that higher ANKRD1 expression was associated with worse clinical outcomes^8^. However, our results did not demonstrate a significant correlation (P > 0.05). Hence, the role of ANKRD1 in pan-cancer is likely to be complex and warrants further investigation, particularly in diverse racial or ethnic populations.

DNA hypermethylation in the promoter region usually results in gene silencing. It has been reported that various tumor suppressor genes are silenced by DNA methylation in different types of tumors^15^. Jimenez et al. found that ANKRD1 is inactivated by DNA methylation in several tumor cell lines, including A427 (lung cancer), LNCaP (prostate cancer), MCF7 (breast cancer), and MeWo (skin cancer)^16^. Another study also observed a similar phenomenon in BxPC-3 cell (pancreatic cancer)^17^. However, our results only showed decreased DNA methylation of ANKRD1 in certain tumors, such as BRCA, LUAD, LUSC, and PAAD (Fig. 4A). Interestingly, high expression of ANKRD1 did not demonstrate a protective effect in pan-cancer. Previous research has suggested that the tumor-suppressive effect of ANKRD1 depends on the presence of p53^16^, which is the most frequently mutated gene in human cancer^18^. It remains to be explored whether mutant p53, known as a guardian of cancer cells, reverses the anti-tumor effect of ANKRD1. Furthermore, a large number of microRNAs, which are involved in post-transcriptional regulation of gene expression, have been reported to be dysfunctional in cancer pathogenesis^19^. For instance, Yin, P et al. discovered that miR-3614-5p inhibits ANKRD1, thereby suppressing osteosarcoma cell proliferation and invasion^20^. According to the GSCALite and ENCORI databases, miR-10a-5p, miR-10b-5p, miR-28-3p, and miR-425-5p, which have been implicated in various cancers^21–23^, are predicted to target ANKRD1.

Our drug sensitivity analysis revealed a correlation between ANKRD1 expression and the IC50 of certain chemotherapy drugs and molecular targeted drugs (Fig. 7). Specifically, we observed that low expression of ANKRD1 was associated with increased sensitivity to most of the identified drugs. Lei, Y et al. found that overexpression of ANKRD1 attenuated cisplatin-induced cytotoxicity in ovarian cancer cell lines^9^. Similarly, another study focusing on EGFR-mutant lung cancer observed elevated levels of ANKRD1 in tumor tissues that had failed EGFR-TKI therapy. It was further demonstrated that imatinib could restore the pro-apoptotic activity of afatinib and osimertinib in EGFR-TKI-resistant cells by inhibiting ANKRD1 expression^10^. Based on these findings, we speculate that decreasing ANKRD1 expression or function may be a potential strategy to mitigate tumor drug resistance.

Immune cell infiltration analysis revealed a strong association between ANKRD1 expression and cancer-associated fibroblast and macrophage, particularly M2 macrophage. Previous studies have confirmed the critical role of the cancer-associated fibroblast in tumor immune escape, immunosuppressive tumor microenvironment, extracellular matrix remodeling, and chemoresistance^24^. M2 macrophages, traditionally considered pro-tumorigenic, are closely related to anti-inflammatory, angiogenic, and immune suppression^25^. It would be interesting to investigate whether ANKRD1 influences immune infiltrates, leading to different survival outcomes. The close correlation between ANKRD1 expression and several known immune checkpoints, especially in LUSC (Fig. 9), further highlights the potential role ANKRD1 in immune regulation. Moreover, GO and KEGG pathway analysis of ANKRD1 in COAD, GBM, LUSC, and STAD indicate that ANKRD1 may impact inflammatory and immune responses. A recent study found that ANKRD1 can be upregulated by tumor necrosis factor alpha (TNFα) and inhibit the transcriptional activity of nuclear factor-kappa B (NF-κB)^26^, providing evidence that for the anti-inflammatory effects of ANKRD1. Interestingly, GO and KEGG pathway analysis of ANKRD1 in HNSC did not enrich any cancer-related signaling pathways, but related to myocardial-related functions or pathways. Increased ANKRD1 expression has been reported in various cardiomyopathies^27^, suggesting its potential as a biomarker for cardiac diseases^27^. Evidently, apart from the tumor itself, treatment side effects (such as drug or radiation-induced heart damage), underlying diseases (diabetes, hypertension, and chronic renal function failure), and other non-tumorous factors also significantly impact the prognosis of cancer patients. Additionally, our recent research found that serum ANKRD1 can predict the cardiotoxicity induced by anthracycline treatment in acute lymphoblastic leukemia^28^. Therefore, ANKRD1 may influence the prognosis of patients with HNSC by affecting myocardial functions.

Furthermore, in vitro experiments conducted with CRC cells regarded ANKRD1 as an oncogene. ANKRD1 may promote the migration, and invasion of COAD via inhibiting apoptosis in CRC cells. Additionally, ANKRD1, known as a stress-response protein^27^, may enhance the adaptability of tumor cells to their microenvironment. However, it is important to note that this study has certain limitations. Firstly, due to considerable heterogeneity among different populations, some inconsistent findings were observed between this study and the reported cohort study. Therefore, to confirm the predictive effect of ANKRD1, a larger sample size encompassing diverse racial groups would be required.

## Conclusion

ANKRD1 shows potential as a diagnostic or prognostic biomarker in pan-cancer, especially COAD. Additionally, we speculated that ANKRD1 may play a role in tumor drug resistance via inflammatory and immune-related pathways. Alternatively, it might impact cancer patient prognosis through non-tumorous pathways as a gene associated with myocardial function. Therefore, further research is warranted to confirm the findings of ANKRD1 in pan-cancer.

## Materials and methods

### Data source and processing

We collected ANKRD1 data, including mRNA expression, DNA methylation, somatic mutation, copy number variant (CNV) data, and clinical follow-up information, from the Cancer Genome Atlas (TCGA, https://portal.gdc.cancer.gov/) database. Our dataset comprised over 10,000 patients with 33 different types of cancers. To supplement the limited normal samples, we also obtained gene expression data from normal tissues in the Genotype-Tissue Expression (GTEx, http://gtexportal.org/) database. In order to normalize the mRNA expression data, we converted them to log2^(TPM+1)^ using R language. For samples with duplicates, we calculated the average mRNA expression level. The specific numbers of cases evaluated for each tumor type in our study are provided in Table S1.

### miRNA regulation analysis

The miRNA regulation analysis of ANKRD1 in different cancers was explored using two databases: GSCALite (http://bioinfo.life.hust.edu.cn/web/GSCALite/)^29^ and the Encyclopedia of RNA Interactomes (ENCORI, https://starbase.sysu.edu.cn/index.php)^30^ databases.

### Drug sensitivity analysis

The IC50 data of drugs and gene expression data were obtained from the Cancer Therapeutics Response Portal (CTRP) database. Only FDA-approved drugs were included in the analysis.

### Functional enrichment analysis

We conducted Gene Ontology (GO) analysis and Kyoto Encyclopedia of Genes and Genomes (KEGG) pathway^31^ analysis by uploading the top 300 most relevant genes of ANKRD1 to the Database for Annotation, Visualization, and Integrated Discovery (DAVID, https://david.ncifcrf.gov/)^32^. The results were then displayed in ascending order of the P-value (P < 0.05), showing the top five outcomes.

### Gene set variation analysis (GSVA) and Gene Set Enrichment Analysis (GSEA)

To determine the enrichment score of the enriched BP, MF, and signaling pathways mentioned above, GSVA and GSEA were conducted using the given package (R environment). The relevant gene sets were obtained from the AmiGO 2 portal (http://amigo.geneontology.org/amigo) and the KEGG database.

### Cell culture

The human colorectal cancer cell lines (Caco2 and SW480) were obtained from ATCC (American Type Culture Collection, Manassas, VA, USA). Both cell lines were confirmed using STR profiling. The cells were cultured in RPMI 1640 (Caco2) or Dulbecco’s Modified Eagle Medium (DMEM) (SW480), supplemented with 10% FBS and 1% penicillin/streptomycin, in a 37 °C incubator with 5% CO_2_.

### Plasmid construction, transfection, and transduction

The ANKRD1 knockout and overexpression plasmids were purchased from Beyotime Biotechnology (Nanjing, Jiangsu, China) and VectorBuilder (Guangzhou, Guangdong, China), respectively. These recombinant lentiviral plasmids were expressed in 293 T cells, and lipofectamine 3000 transfection reagent (Invitrogen, CA, USA) was used to enhance the efficiency of virus transfection. The viruses overexpressing ANKRD1 were labeled as ANKRD1 OE, while the viruses silencing ANKRD1 were designated as ANKRD1 KO. After three days, the cancer cells were transduced with the recombinant lentivirus or blank pLVX-Puro and pLenti-Control-sgRNA (negative control). Following 72 h, complete medium containing 2 μg/ml puromycin was added to establish stable cell lines.

### Real-time quantitative polymerase chain reaction (qPCR)

Total RNA was extracted from Caco2 and SW480 cell lines using TRIzol reagent (Sangon Biotech, Shanghai, China), followed by reverse transcription into cDNA using a Prime Script RT Master Mix (Takara, Japan). Subsequently, qPCR was conducted using a 2× SYBR Green qPCR Mix (Takara, Japan) to detect the levels of the targeted mRNA, with GAPDH serving as the reference gene. The primer sequences used were as follows: ANKRD1 Forward primer: 5′-ATGTGGCGGTGAGGACTGG-3′, ANKRD1 Reverse primer: 5′-GTCGGATCATCTTATAGCGGTTCAG-3′, GAPDH Forward primer: 5′-ACCCACTCCTCCACCTTTGAC-3′, GAPDH Reverse primer: 5′-TCCACCACCCTGTTGCTGTAG-3′.

### Western blotting (WB)

The total protein was extracted using lysis buffer and quantified using a BCA protein assay (Beyotime, China). The protein samples were then mixed with 4× loading buffer and heated at 100 ℃ for 5 min. Subsequently, the samples were separated by SDS-PAGE, and transferred onto a PVDF membrane. Thereafter, the membrane was blocked with 5% skim milk at room temperature for 1 h. Then, the blots were incubated overnight at 4 ℃ with primary antibodies against CARP (1:250, Santa Cruz, sc-365056), Bax (1:1000, Abcam, ab7977), cleared Caspase 3 (1:1000, CST, 9661s) and β-tublin (1:2000, proteintech, 10094-I-AP). After washing the membrane with PBST (PBS with 0.1% Tween-20), it was incubated with a secondary antibody (1:2000) at room temperature for 1 h, followed by detection using the electrochemiluminescence (ECL) method. The fluorescent signal was captured using a BioRad imaging system (BioRad, CA, USA). All raw data is displayed in the supplementary information (Fig. S4).

### Cell counting kit-8 assay

A total of 2000 cells suspended in 100 µl medium were seeded in each well of a 96-well plate. The test was initiated after 24 h and lasted for 4 consecutive days (1, 2, 3, 4 days). To each well, 10 µl CCK-8 reagent was added. After incubation for 1 h, the absorbance at 450 nm was measured using a microplate reader (BioTek, USA).

### Scratch test

Caco2 and SW480 cells were seeded in 24-well plates. After 24 h, a 200 µl pipette tip was used to create scratches on the cell monolayers, and then 1 ml of serum-free medium was added to each well. Subsequently, images were captured at 0 h and 24 h using a fluorescence microscope (ThermoFisher scientific, USA).

### Colony-formation assay

A total of 2000 cells were seeded in 6-well plates and cultured for 7 days. The cell colonies were then fixed with 4% formaldehyde for 15 min and stained with 0.5% crystal violet before being imaged.

### Transwell migration and invasion assay

Transwell assays were performed using 24-well Transwells (8 μm pore size, Corning, USA). The upper chamber of the transwell chamber was pre-coated with Matrigel matrix glue (Corning Company, USA; matrix glue: serum-free medium = 1:4) for cell invasion assays, but not for cell migration assays. In the upper chamber, a total of 25 thousand cells were seeded in serum-free medium. After 48 h of incubation at 37 °C, the cells in the chamber were fixed with 4% paraformaldehyde for 15 min and stained with 10% crystal violet for 5 min. Then, cells on the top surface of the chamber were removed using cotton swabs. The number of migrated cells was imaged.

### Statistical analysis

The statistical analyzes and visualization mentioned above were performed using R (version 4.2.3) and SPSS 21.0 software. An independent sample T test or one-way ANOVA was used to compare two or more than two groups, respectively. Pearson correlation analysis was conducted to assess the correlation between the two groups. COX regression analysis and Kaplan–Meier (K-M) curve were applied to evaluate the prognostic value. A P-value < 0.05 was considered statistically significant.

### Supplementary Information

Supplementary Information.

## Data Availability

The datasets analysed in this study are available in several databases. These include the Cancer Genome Atlas (TCGA, https://portal.gdc.cancer.gov/) database, the Genotype-Tissue Expression (GTEx, http://gtexportal.org/) database, the GSCALite (http://bioinfo.life.hust.edu.cn/web/GSCALite/) database, the Encyclopedia of RNA Interactomes (ENCORI, https://starbase.sysu.edu.cn/index.php) database, the CTRP (The Cancer Therapeutics Response Portal) database, the Database for Annotation, Visualization, and Integrated Discovery (DAVID, https://david.ncifcrf.gov/) database, Kyoto Encyclopedia of Genes and Genomes (KEGG, https://www.kegg.jp/kegg/kegg1.html), AmiGO 2 portal (http://amigo.geneontology.org/amigo). All data is publicly available.
