# Supplementary material for: Pan-cancer integrated analysis of ANKRD1 expression, prognostic value, and potential implications in cancer
Source: Sci Rep. 2024 Mar 4;14:5268. doi: 10.1038/s41598-024-56105-2 (PMC10912109; doi:10.1038/s41598-024-56105-2)

Supplemental Information

Table S1. Demographic details in TCGA and GTEX groups

Abbr	Cancer types	TCGA (n=10560) normal* (n=730)	TCGA (n=10560) tumor (n=9830)	TCGA+GTEX (n=17152) normal& (n=7322)	TCGA+GTEX (n=17152) tumor (n=9830)
ACC	Adrenocortical carcinoma	-	79	128	79
BLCA	Bladder Urothelial Carcinoma	19	408	28	408
BRCA	Breast invasive carcinoma	113	1091	292	1091
CESC	Cervical squamous cell carcinoma and endocervical adenocarcinoma	3	304	13	304
CHOL	Cholangiocarcinoma	9	36	9	36
COAD	Colon adenocarcinoma	41	456	349	456
DLBC	Lymphoid Neoplasm Diffuse Large B-cell Lymphoma	-	48	-	48
ESCA	Esophageal carcinoma	11	161	664	161
GBM	Glioblastoma multiforme	5	154	1157	154
HNSC	Head and Neck squamous cell carcinoma	44	500	44	500
KICH	Kidney Chromophobe	24	65	52	65
KIRC	Kidney renal clear cell carcinoma	72	530	100	530
KIRP	Kidney renal papillary cell carcinoma	32	288	60	288
LAML	Acute Myeloid Leukemia	-	151	70	151
LGG	Brain Lower Grade Glioma	-	511	1152	511
LIHC	Liver hepatocellular carcinoma	50	371	160	371
LUAD	Lung adenocarcinoma	59	513	347	513
LUSC	Lung squamous cell carcinoma	49	501	337	501
MESO	Mesothelioma	-	86	-	86
OV	Ovarian serous cystadenocarcinoma	-	374	88	374
PAAD	Pancreatic adenocarcinoma	4	177	171	177
PCPG	Pheochromocytoma and Paraganglioma	3	178	131	178
PRAD	Prostate adenocarcinoma	52	495	152	495
READ	Rectum adenocarcinoma	10	166	102	166
SARC	Sarcoma	2	259	2	259
SKCM	Skin Cutaneous Melanoma	1	103	813	103
STAD	Stomach adenocarcinoma	32	375	206	375
TGCT	Testicular Germ Cell Tumors	-	150	165	150
THCA	Thyroid carcinoma	58	502	337	502
THYM	Thymoma	2	119	2	119

UCEC	Uterine Corpus Endometrial Carcinoma	35	543	113	543
UCS	Uterine Carcinosarcoma	-	56	78	56
UVM	Uveal Melanoma	-	80	-	80

Abbr: abbreviation; * paracancerous tissues; & paracancerous tissues from TCGA database and normal tissues from GTEX database.

Table S2. Gene Set Enrichment Analysis (GSEA) of ANKRD1 in pan-cancer

cancer type	Ontology	ID	Description	set size	ES	NES	P value	FDR
COAD	BP	GO:0002683	negative regulation of immune system process	424	0.54	1.97	1.00E-10	2.63E-09
COAD	BP	GO:0007159	leukocyte cell-cell adhesion	394	0.56	2.03	1.00E-10	2.63E-09
COAD	BP	GO:0007389	pattern specification process	450	0.45	1.62	1.48E-10	3.69E-09
COAD	BP	GO:0045785	positive regulation of cell adhesion	462	0.57	2.09	1.00E-10	2.63E-09
COAD	BP	GO:0003002	regionalization	351	0.46	1.65	1.77E-09	3.47E-08
COAD	CC	GO:0062023	collagen-containing extracellular matrix	419	0.64	2.34	1.00E-10	2.63E-09
COAD	CC	GO:0009897	external side of plasma membrane	433	0.61	2.21	1.00E-10	2.63E-09
COAD	CC	GO:0005925	focal adhesion	409	0.46	1.66	1.00E-10	2.63E-09
COAD	CC	GO:0030055	cell-substrate junction	417	0.46	1.66	1.00E-10	2.63E-09
COAD	CC	GO:0030667	secretory granule membrane	298	0.53	1.90	1.00E-10	2.63E-09
COAD	MF	GO:0140375	immune receptor activity	143	0.69	2.39	1.00E-10	2.63E-09
COAD	MF	GO:0019865	immunoglobulin binding	22	0.70	1.92	0.000126	0.000773
COAD	MF	GO:0005178	integrin binding	145	0.67	2.35	1.00E-10	2.63E-09
COAD	MF	GO:0005539	glycosaminoglycan binding	219	0.58	2.08	1.00E-10	2.63E-09
COAD	MF	GO:0038024	cargo receptor activity	78	0.62	2.02	1.86E-08	2.93E-07
COAD	KEGG	hsa05150	Staphylococcus aureus infection	89	0.74	2.49	1.00E-10	1.11E-09
COAD	KEGG	hsa00532	Glycosaminoglycan biosynthesis - chondroitin sulfate / dermatan sulfate	21	0.77	2.09	7.06E-07	4.20E-06
COAD	KEGG	hsa04145	Phagosome	142	0.62	2.16	1.00E-10	1.11E-09
COAD	KEGG	hsa05416	Viral myocarditis	64	0.71	2.32	1.00E-10	1.11E-09
COAD	KEGG	hsa04610	Complement and coagulation cascades	85	0.68	2.26	1.00E-10	1.11E-09
GBM	BP	GO:0043062	extracellular structure organization	304	0.54	2.72	1.00E-10	2.20E-09
GBM	BP	GO:0030198	extracellular matrix organization	303	0.54	2.72	1.00E-10	2.20E-09

GBM	BP	GO:0045229	external encapsulating structure organization	306	0.54	2.71	1.00E-10	2.20E-09
GBM	BP	GO:0050900	leukocyte migration	366	0.55	2.81	1.00E-10	2.20E-09
GBM	BP	GO:0042060	wound healing	417	0.50	2.59	1.00E-10	2.20E-09
GBM	CC	GO:0062023	collagen-containing extracellular matrix	421	0.56	2.90	1.00E-10	2.20E-09
GBM	CC	GO:0098644	complex of collagen trimers	22	0.71	2.24	3.92E-05	0.00027
GBM	CC	GO:0005581	collagen trimer	85	0.61	2.56	1.00E-10	2.20E-09
GBM	CC	GO:0005788	endoplasmic reticulum lumen	299	0.54	2.73	1.00E-10	2.20E-09
GBM	CC	GO:0005604	basement membrane	97	0.52	2.26	2.09E-08	3.07E-07
GBM	MF	GO:0005125	cytokine activity	217	0.56	2.74	1.00E-10	2.20E-09
GBM	MF	GO:0030546	signaling receptor activator activity	454	0.46	2.40	1.00E-10	2.20E-09
GBM	MF	GO:0048018	receptor ligand activity	447	0.47	2.45	1.00E-10	2.20E-09
GBM	MF	GO:0030020	extracellular matrix structural constituent conferring tensile strength	41	0.65	2.38	2.67E-07	3.12E-06
GBM	MF	GO:0005126	cytokine receptor binding	243	0.55	2.72	1.00E-10	2.20E-09
GBM	KEGG	hsa04668	TNF signaling pathway	110	0.64	2.82	1.00E-10	1.78E-09
GBM	KEGG	hsa04657	IL-17 signaling pathway	91	0.65	2.77	1.00E-10	1.78E-09
GBM	KEGG	hsa04060	Cytokine-cytokine receptor interaction	279	0.58	2.88	1.00E-10	1.78E-09
GBM	KEGG	hsa05323	Rheumatoid arthritis	85	0.66	2.81	1.00E-10	1.78E-09
GBM	KEGG	hsa04610	Complement and coagulation cascades	85	0.62	2.61	1.00E-10	1.78E-09
HNSC	BP	GO:0003012	muscle system process	426	0.76	2.71	1.00E-10	2.55E-09
HNSC	BP	GO:0006936	muscle contraction	333	0.78	2.77	1.00E-10	2.55E-09
HNSC	BP	GO:0055001	muscle cell development	181	0.81	2.79	1.00E-10	2.55E-09
HNSC	BP	GO:0051146	striated muscle cell differentiation	275	0.76	2.67	1.00E-10	2.55E-09
HNSC	BP	GO:0042692	muscle cell differentiation	379	0.75	2.63	1.00E-10	2.55E-09
HNSC	CC	GO:0030016	myofibril	229	0.86	2.97	1.00E-10	2.55E-09
HNSC	CC	GO:0043292	contractile fiber	237	0.85	2.96	1.00E-10	2.55E-09

HNSC	CC	GO:0030017	sarcomere	210	0.86	2.97	1.00E-10	2.55E-09
HNSC	CC	GO:0031674	I band	141	0.86	2.90	1.00E-10	2.55E-09
HNSC	CC	GO:0030018	Z disc	129	0.85	2.86	1.00E-10	2.55E-09
HNSC	MF	GO:0003779	actin binding	422	0.72	2.54	1.00E-10	2.55E-09
HNSC	MF	GO:0008307	structural constituent of muscle	41	0.89	2.58	1.00E-10	2.55E-09
HNSC	MF	GO:0051015	actin filament binding	209	0.73	2.52	1.00E-10	2.55E-09
HNSC	MF	GO:0005523	tropomyosin binding	16	0.95	2.28	1.00E-10	2.55E-09
HNSC	MF	GO:0005516	calmodulin binding	196	0.62	2.13	1.00E-10	2.55E-09
HNSC	KEGG	hsa05414	Dilated cardiomyopathy	101	0.82	2.68	1.00E-10	1.69E-09
HNSC	KEGG	hsa04261	Adrenergic signaling in cardiomyocytes	152	0.73	2.46	1.00E-10	1.69E-09
HNSC	KEGG	hsa04260	Cardiac muscle contraction	81	0.76	2.44	1.00E-10	1.69E-09
HNSC	KEGG	hsa05410	Hypertrophic cardiomyopathy	95	0.83	2.68	1.00E-10	1.69E-09
HNSC	KEGG	hsa05412	Arrhythmogenic right ventricular cardiomyopathy	83	0.80	2.54	1.00E-10	1.69E-09
LUSC	BP	GO:0043129	surfactant homeostasis	14	0.81	1.84	0.000132	0.000764
LUSC	BP	GO:0048762	mesenchymal cell differentiation	246	0.47	1.55	1.36E-05	0.000105
LUSC	BP	GO:0006959	humoral immune response	287	0.58	1.91	1.00E-10	2.49E-09
LUSC	BP	GO:0006957	complement activation, alternative pathway	17	0.78	1.88	6.06E-05	0.000389
LUSC	BP	GO:0048875	chemical homeostasis within a tissue	16	0.79	1.86	8.68E-05	0.000537
LUSC	CC	GO:0042599	lamellar body	17	0.80	1.91	2.37E-05	0.000173
LUSC	CC	GO:0005771	multivesicular body	62	0.66	1.94	2.03E-07	2.49E-06
LUSC	CC	GO:0030139	endocytic vesicle	331	0.63	2.09	1.00E-10	2.49E-09
LUSC	CC	GO:0045334	clathrin-coated endocytic vesicle	86	0.65	2.00	1.70E-09	3.17E-08
LUSC	CC	GO:0030136	clathrin-coated vesicle	202	0.52	1.70	2.08E-07	2.54E-06
LUSC	MF	GO:0038024	cargo receptor activity	78	0.67	2.04	2.64E-10	6.05E-09
LUSC	MF	GO:0005044	scavenger receptor activity	45	0.73	2.06	1.65E-08	2.55E-07

LUSC	MF	GO:0140375	immune receptor activity	143	0.75	2.41	1.00E-10	2.49E-09
LUSC	MF	GO:0030246	carbohydrate binding	258	0.58	1.92	1.00E-10	2.49E-09
LUSC	MF	GO:0019199	transmembrane receptor protein kinase activity	77	0.51	1.55	0.00232	0.008471
LUSC	KEGG	hsa04610	Complement and coagulation cascades	85	0.74	2.27	1.00E-10	1.18E-09
STAD	BP	GO:0007608	sensory perception of smell	406	0.58	2.05	1.00E-10	5.80E-08
STAD	BP	GO:0051918	negative regulation of fibrinolysis	13	0.76	1.94	6.41E-05	0.002463
STAD	BP	GO:0050911	detection of chemical stimulus involved in sensory perception of smell	382	0.59	2.07	1.00E-10	5.80E-08
STAD	KEGG	hsa04610	Complement and coagulation cascades	85	0.46	1.51	0.000354	0.002207
STAD	KEGG	hsa04740	Olfactory transduction	395	0.57	2.01	1.00E-10	4.05E-09

COAD, Colon adenocarcinoma; GBM, Glioblastoma multiforme; HNSC, Head and Neck squamous cell carcinoma; LUSC, Lung squamous cell carcinoma; STAD, Stomach adenocarcinoma; BP, biological process; CC, cellular component; MF, molecular function; ES, Enrichment score.

Figure S1.

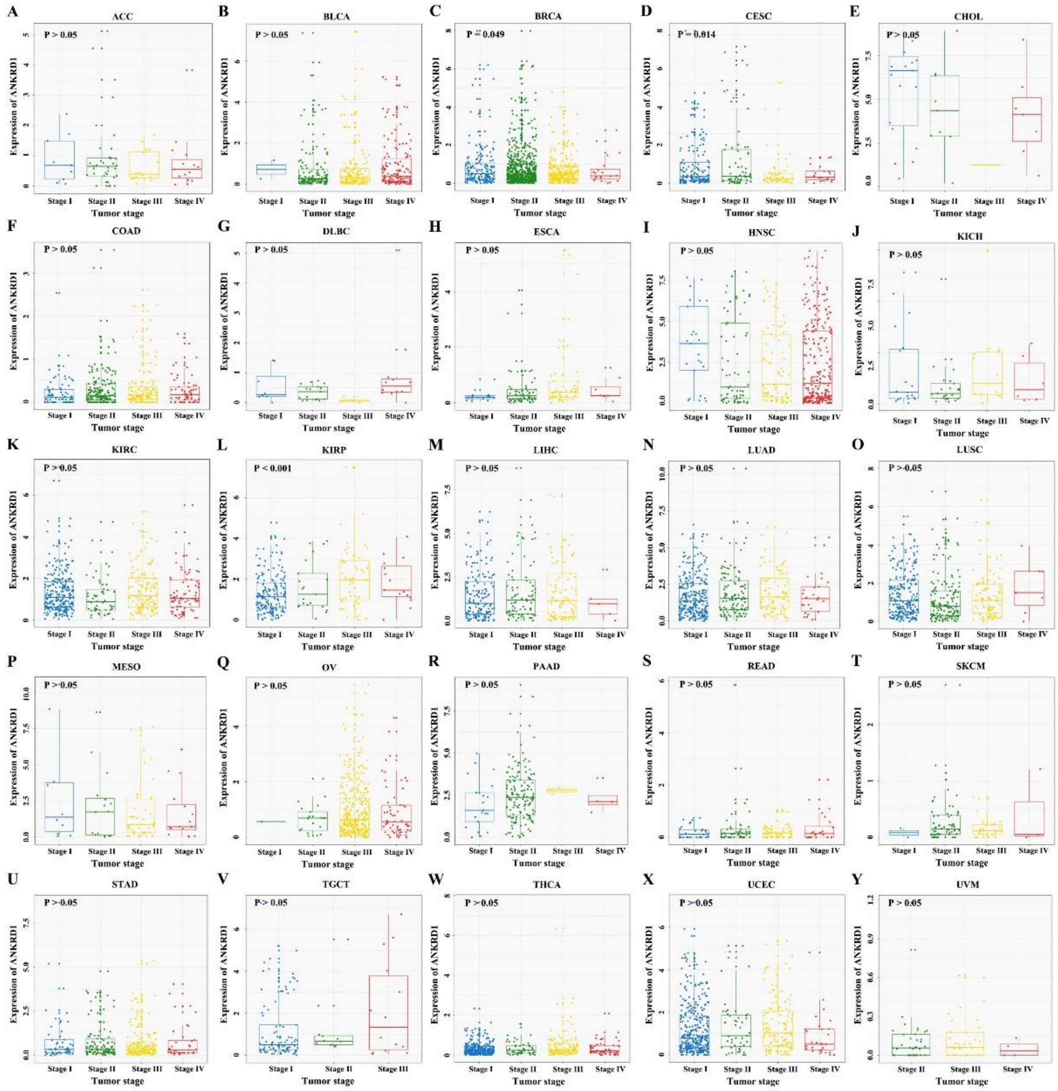

Figure S1. ANKRD1 expression levels in tumor stages of pan-cancer.

Figure S2.

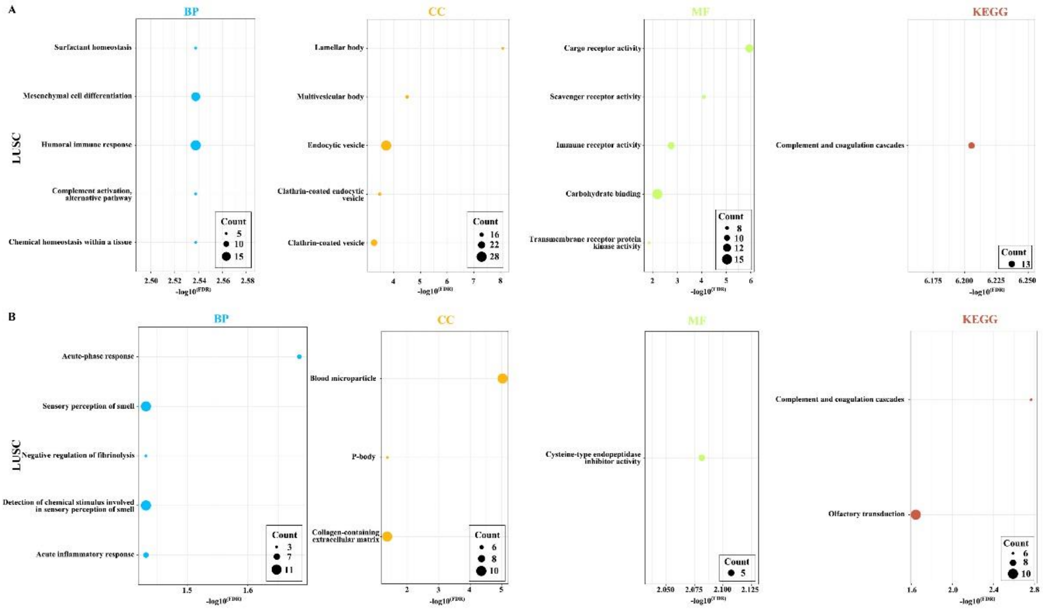

Figure S2. GO and KEGG enrichment analyses for ANKRD1. Top 5 pathways enriched in the BP, CC, MF, and KEGG analyses in (A) LUSC, and (B) STAD.

Figure S3.

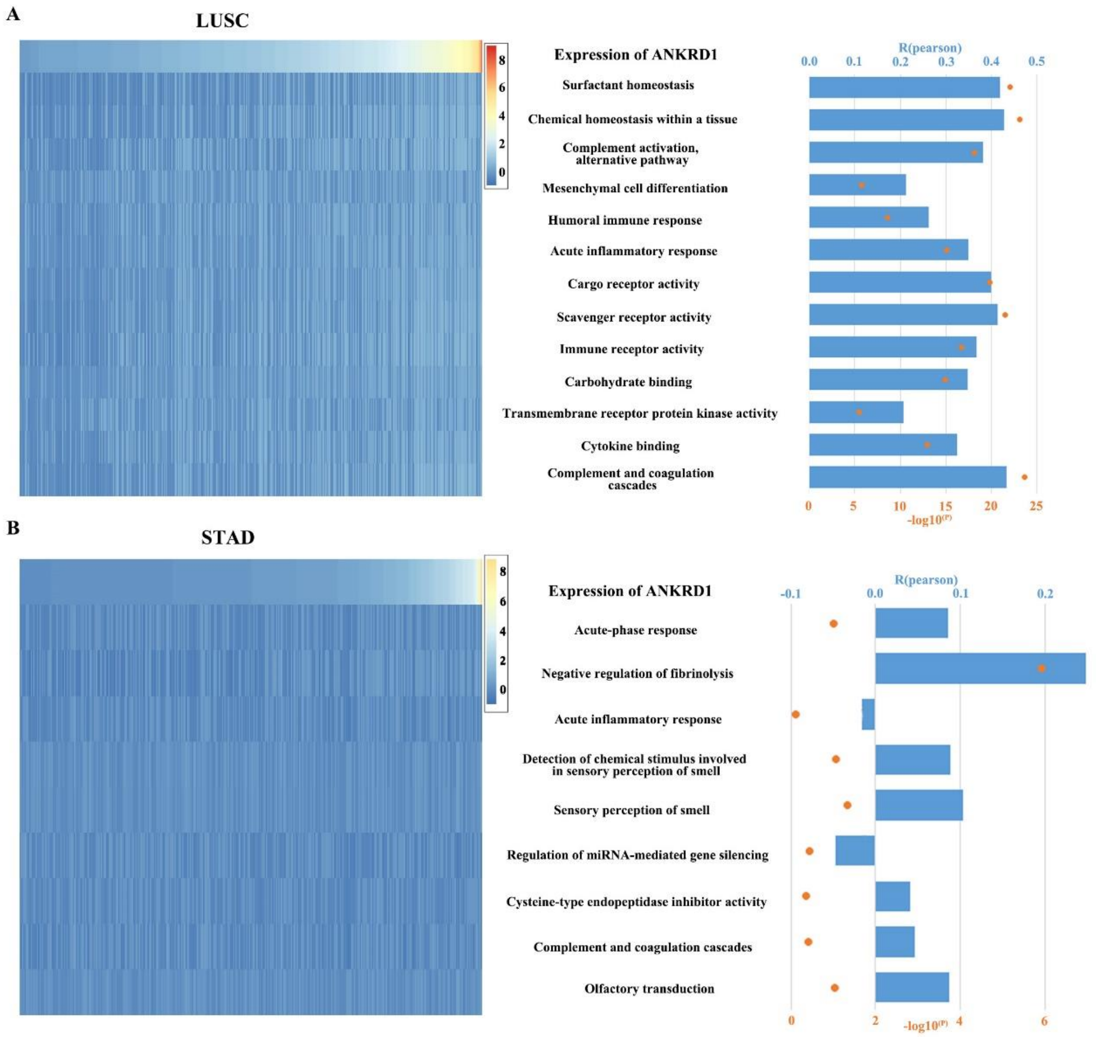

Figure S3. Correlation analysis between ANKRD1 expression and the enrichment scores of enriched pathways based on the BP, MF, and KEGG analyses in (A) LUSC, and (B) STAD. The heatmap showed ANKRD1 mRNA expression and the enrichment scores of each patient in the TCGA database. The samples were arranged in ascending order of the expression of ANKRD1. The column graph and line graph on the right showed the R-value and P-value of the correlation analysis.

Figure S4 **WB original data**

Figure 13B Caco2

β-tublin

Control ANKRD1 KO

60KD

45KD

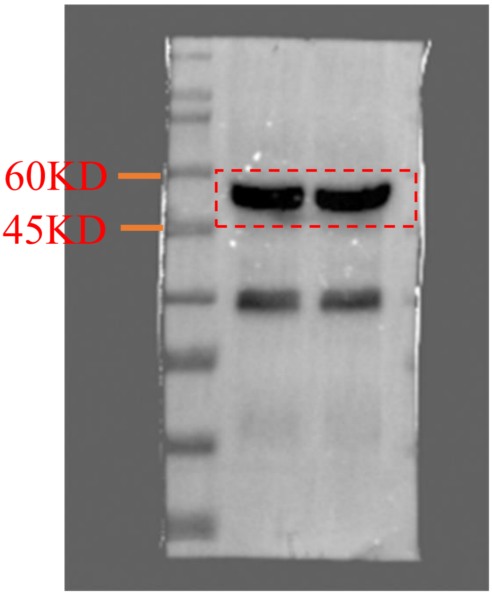

ANKRD1

Control ANKRD1 KO

45KD

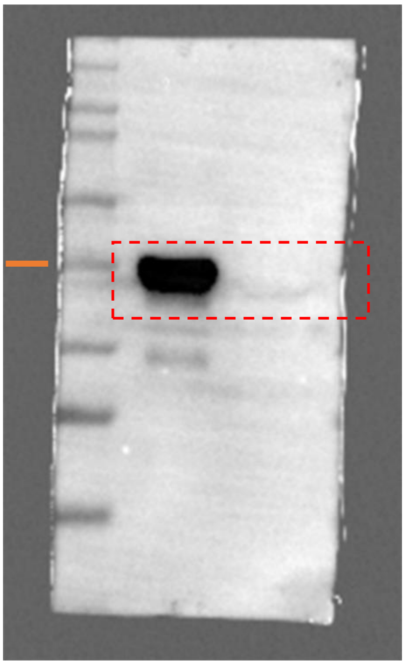

cleared Caspase 3

Control ANKRD1 KO

25KD

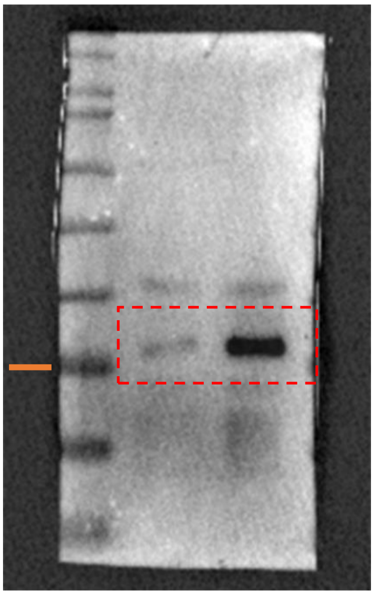

Bax

Control ANKRD1 KO

25KD

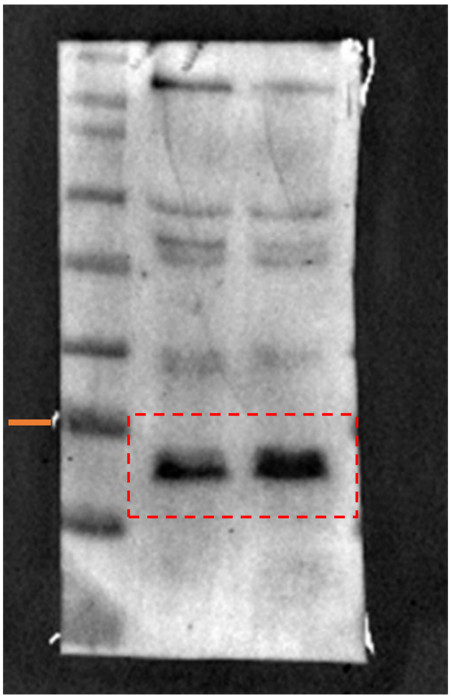

Figure 13C SW480

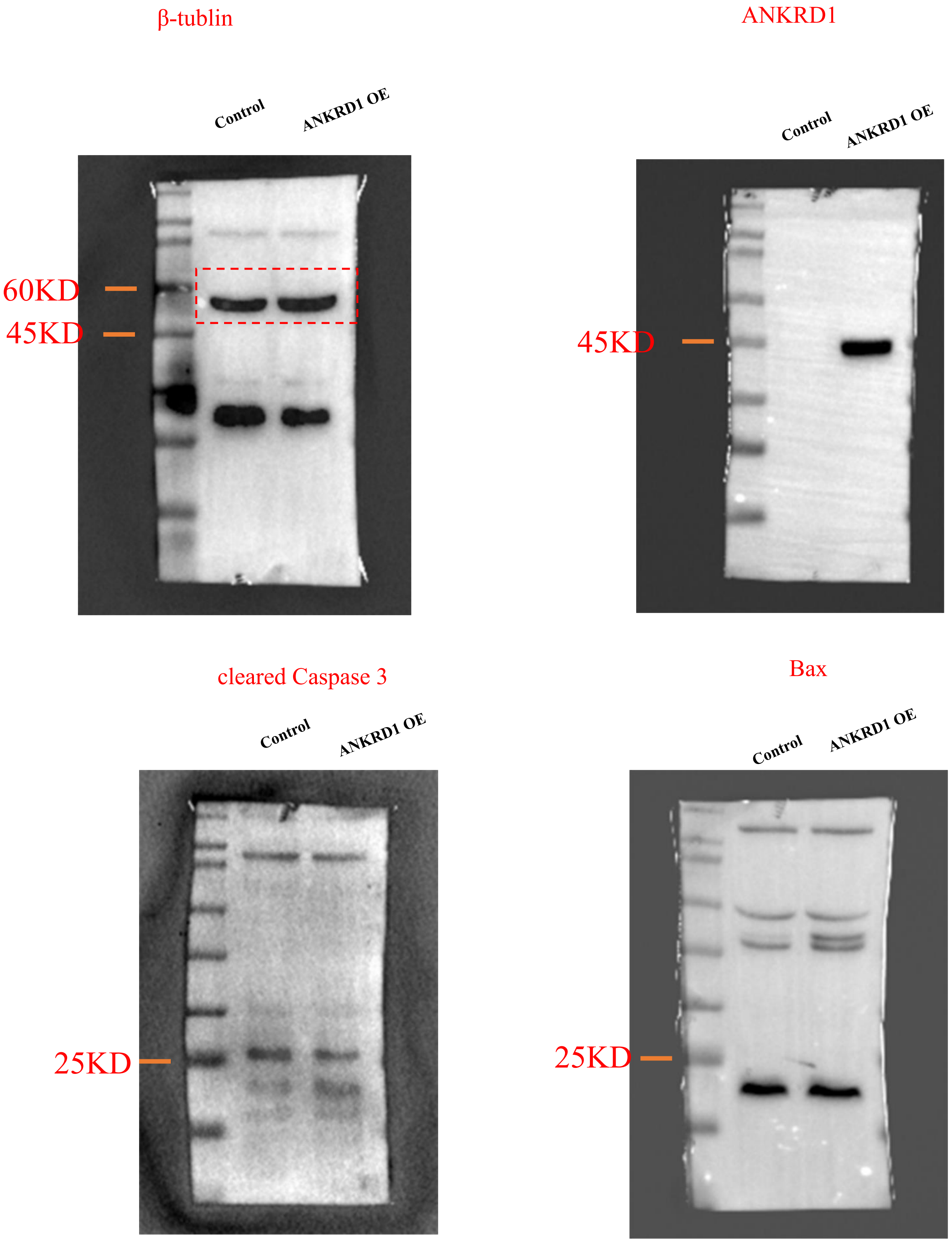

Supplement: Supplementary file 1 — Supplementary Information. [file 41598_2024_56105_MOESM1_ESM.pdf]
